# Catalytic Upgrading of Acetaldehyde to Acetoin Using a Supported N‐Heterocyclic Carbene Catalyst

**DOI:** 10.1002/cssc.202400647

**Published:** 2024-08-08

**Authors:** Maurice Belleflamme, Jerome Hommes, Riza Dervisoglu, Ettore Bartalucci, Thomas Wiegand, Anna Katharina Beine, Walter Leitner, Andreas J. Vorholt

**Affiliations:** ^1^ Max Planck Institute for Chemical Energy Conversion Stiftstraße 34–36 45470 Mülheim an der Ruhr Germany; ^2^ Institute for Technical and Macromolecular Chemistry RWTH Aachen University Worringerweg 2 52074 Aachen Germany; ^3^ Department for Biochemical and Chemical Engineering, Laboratory of Industrial Chemistry TU Dortmund University Emil-Figge-Str. 66 44227 Dortmund Germany; ^4^ Department of Mechanical Engineering University of Siegen Paul-Bonatz-Str. 9–11 57076 Siegen Germany

**Keywords:** Catalysis, *N*-heterocyclic carbenes, catalytic upgrading, organocatalysis, solid-state NMR spectroscopy

## Abstract

We report the catalytic synthesis of 3‐hydroxy‐2‐butanon (acetoin) from acetaldehyde as a key step in the synthesis of C_4_‐molecules from ethanol. Facile C−C bond formation at the α‐carbon of the C_2_ building block is achieved using an *N*‐heterocyclic carbene (NHC) catalyst. The immobilization of the catalyst on a *Merrifield*’s peptide resin and its spectroscopic characterisation using solid‐state Nuclear Magnetic Resonance (NMR) is described herein. The immobilization of the NHC catalyst allows for process intensification steps and the reported catalytic system was subjected to batch recycling as well as continuous flow experiments. The robustness of the catalytic system was shown over a maximum of 10 h time‐on‐stream. Overall, high selectivity *S*>90 % was observed. The observed deactivation of the catalyst with increasing time‐on‐stream is explained by *ex‐situ* ^1^H solution‐state, as well as ^13^C and ^15^N solid‐state NMR spectra allowing us to develop a deeper understanding of the underlying decomposition mechanism of the catalyst.

## Graphical abstract

Discover our innovative synthesis of 3‐hydroxy‐2‐butanone (acetoin) from ethanol‐derived acetaldehyde, a key step in producing C_4_ molecules from bio‐based resources. Using an *N‐*heterocyclic carbene (NHC) catalyst, this process offers efficient C−C bond formation with high selectivity and robust performance. Explore catalyst immobilization, recycling, continuous flow experiments, and deactivation insights from advanced NMR spectroscopy.

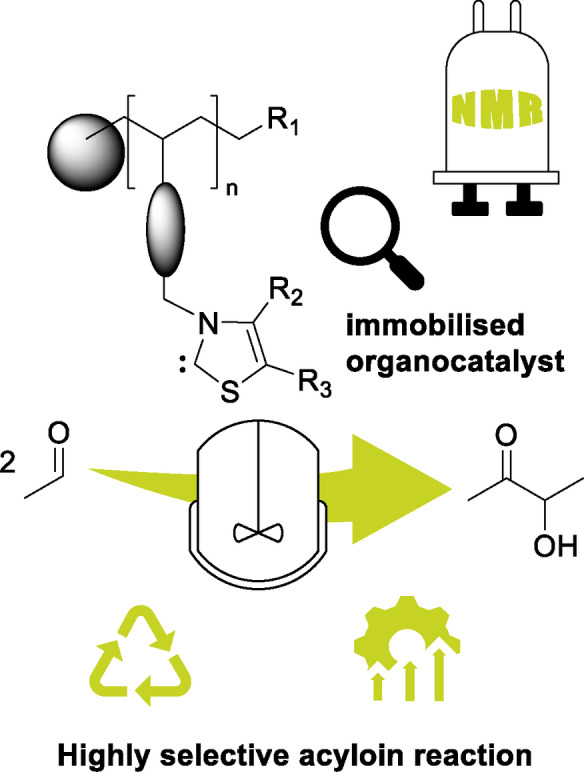

## Introduction

**Scheme 1:**
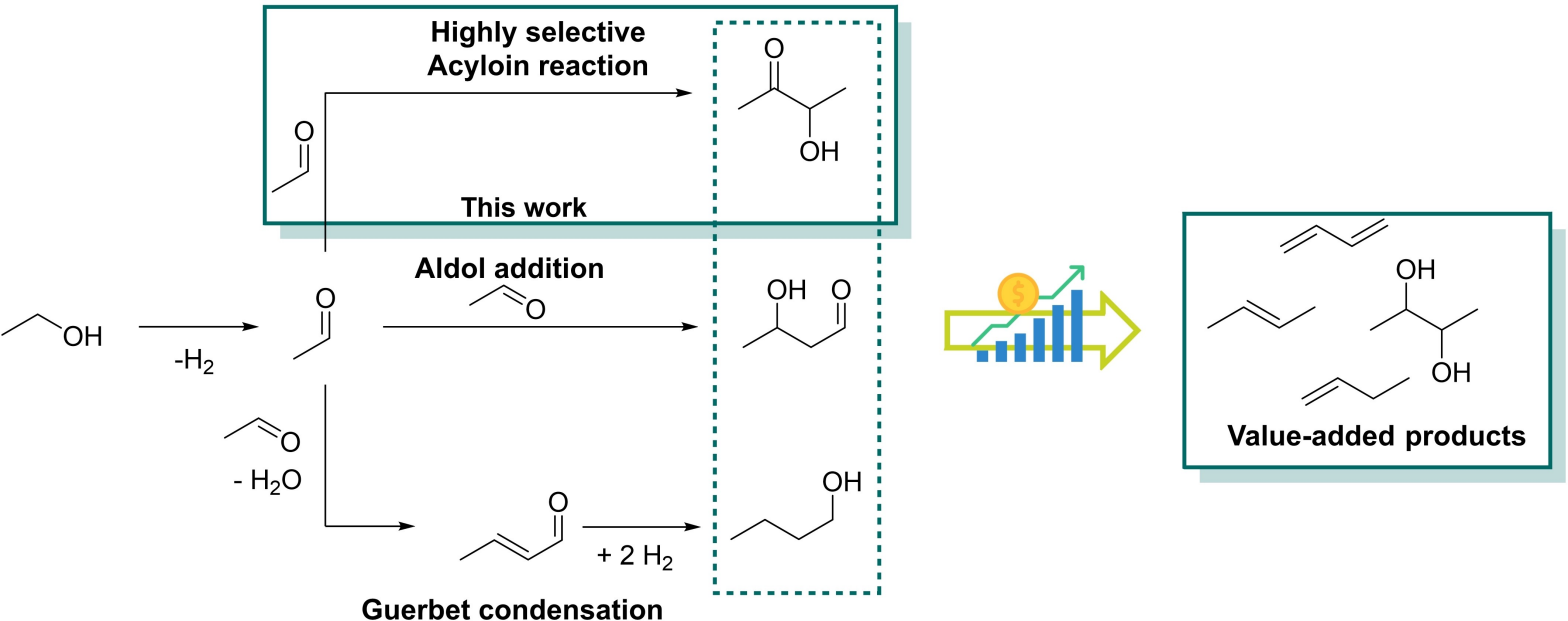
Schematic overview of the possible pathways to upgrade ethanol to C_4_ products that can subsequently be converted to relevant value‐added products for the chemical valuechain.

Using bio‐based raw materials and implementing them in the chemicalvalue chain is of significant importance to reduce the greenhouse gas emission as well as the carbon footprint of the chemical industry.^[1]^ Among the various approaches, using ethanol as bio‐based raw material is particularly interesting due to its great availability and the well‐established large‐scale industrial production processes.[^2^, ^3^, ^4^] However, in the catalytic upgrading of ethanol, the major challenge is to effectively facilitate C−C bond formation between two ethanol molecules (Scheme 1).

In general, upgrading of ethanol can proceed *via* different pathways beginning with the dehydrogenation of ethanol to acetaldehyde. The most common pathways are aldol‐type or *Guerbet* reactions.[^5^, ^6^, ^7^] With the optimal homogeneous catalyst, *Guerbet*‐type reactions can offer a high selectivity towards the desired *n*‐butanol.^[8]^ But even with the most advanced molecular catalysts, the *Guerbet*‐type upgrading of ethanol still suffers from low conversions.[^9^, ^10^] On the other hand, heterogeneously catalysed processes require harsh conditions and suffer from a low selectivity.[^11^, ^12^] In general, ethanol is not the most suitable substrate for the *Guerbet*‐type reaction since the dehydrogenation of ethanol is thermodynamically unfavoured and the selective aldol condensation of acetaldehyde is often challenging.[^5^, ^8^, ^13^] When it comes to aldol‐type addition of aldehydes, the mechanism, reaction and substrate scope is well‐studied.[^14^, ^15^, ^16^] When using acetaldehyde as substrate, achieving a high selectivity towards the desired aldol condensation product is challenging as acetaldehyde has a high tendency to form undesired higher molecular weight side‐products in basic environment.^[17]^ Even if acetaldehyde is used as nucleophile in (crossed) aldol reactions, the reaction is usually mediated by an organocatalyst. When using proline as mediator/catalyst in the self‐aldol reaction of acetaldehyde, very low yields were obtained.^[18]^

A milder and more selective approach to couple aldehydes can be facilitated by using *N*‐heterocyclic carbenes (NHC). The reaction and its catalysts are widely studied, especially for aromatic aldehydes and the mechanism of the reaction was disclosed by *Breslow* in 1958.[^19^, ^20^, ^21^] However, in literature, the reaction is exclusively applied for aromatic aldehydes or longer‐chain alkyl aldehydes. To some extent, NHC catalysed acyloin tandem‐reactions are reported in literature that use higher alcohols.^[22]^ NHC catalysed acetaldehyde self‐addition to 3‐hydroxy‐2‐butanone (acetoin) has remained nearly unexploited.[^23^, ^24^]

In the catalytic upgrading of bio‐ethanol to C_4_ platform chemicals like butenes and 2,3‐butanediol, the selective self‐addition of acetaldehyde to acetoin is a key step, as the industrial dehydration of (bio) ethanol to acetaldehyde is already applied on large scale.[^24^, ^25^, ^26^] For the acetaldehyde self‐coupling reaction to form acetoin it has already been shown that NHC‐based catalysts offer efficient and to some extend even recyclable approaches: *Zhang et al*. reported a homogeneous thiazolium salt that could be used alongside a combination of solid bases for up to three cycles.[^23^, ^24^] Along the recycling pathway, yields towards acetoin decreased from 98 % to 55 %. On the other hand, polystyrene immobilised thiazolium salts in combination with K_2_CO_3_ for example, did not lead to satisfactory results (95 % acetoin yield in the first run, <10 % in the second).^[23]^ To apply this reaction in a bigger scale the total turnover number (TTON) of the catalyst has to be optimized further and the catalytic system has to be more stable while being selective.

In order to perform the ethanol‐derived acetaldehyde to acetoin reaction, a heterogeneous catalyst has to be developed that is highly recyclable and suitable for continuous flow applications. Furthermore, homo‐acyloin reactions reported in literature are not carried out in the corresponding alcohol as solvent. In this case, carrying out the reaction in ethanol as corresponding alcohol would be highly desired. This opens up the possibility of developing an integrated reaction system where acyloin compounds can be directly obtained from alcohols in two reaction steps without purification of the aldehyde and removal of excess alcohol after the first reaction step. Lastly, to the best of our knowledge, there is no in‐depth study of the catalyst and the structural changes it undergoes.

## Results and Discussion

**Figure 1:**
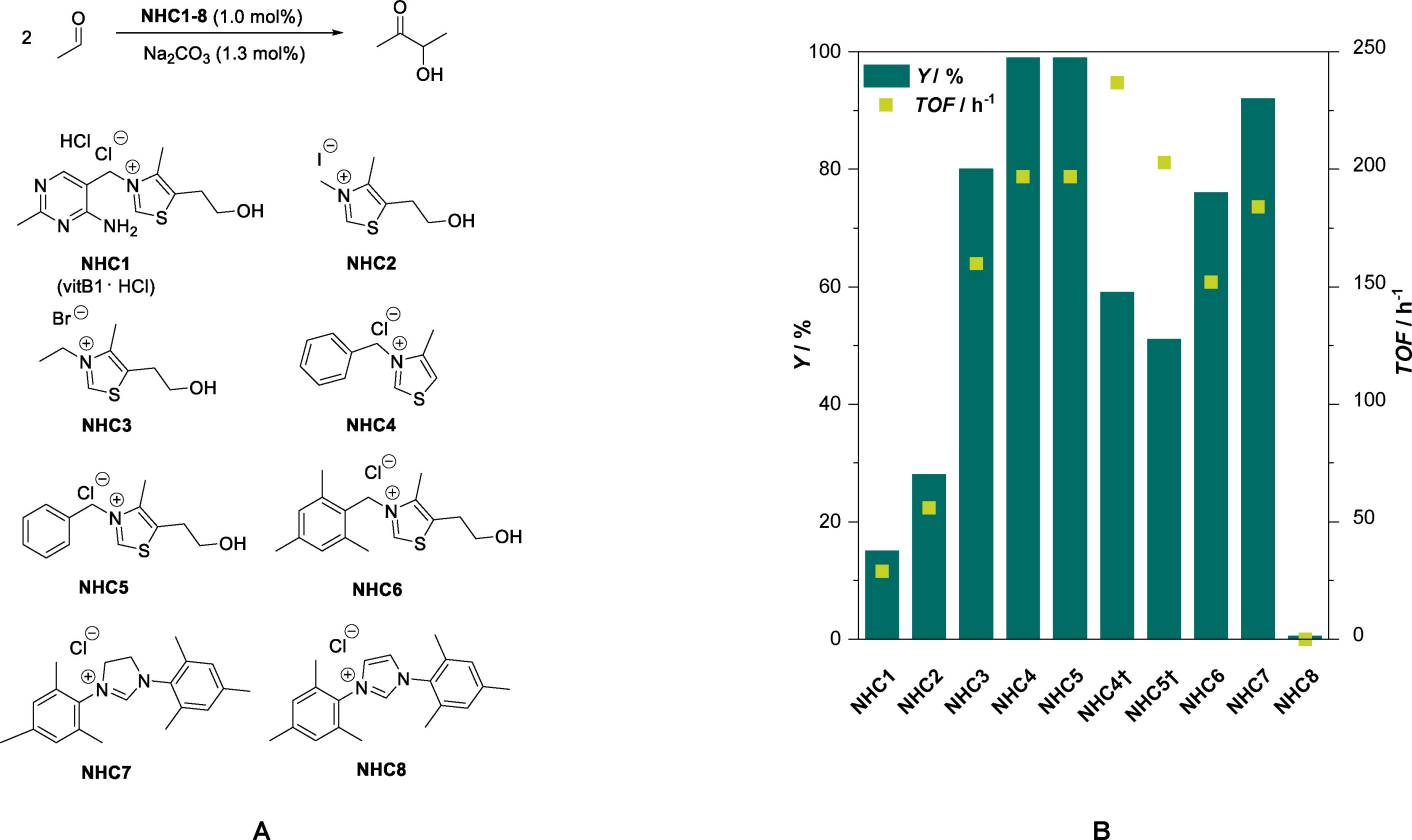
**A**: Overview of the *N*‐heterocyclic carbene catalyst precursors screened in the acetaldehyde self‐addition. **B**: Yield (*Y*) and turn‐over‐frequency (*TOF*) obtained for the various NHC precursors **NHC1**‐**8**. Reaction conditions: *V*(acetaldehyde)=2.0 mL, *n*(acetaldehyde)=36 mmol, *c*(NHC)=1.0 mol%, *c*(Na_2_CO_3_)=1.3 mol%, *T*=100 °C, *t*=30 min, yields determined from GC‐FID using mesitylene as an internal standard. †: *t*=15 min at otherwise identical conditions

Motivated by the reports of *Zhang et al*.[^23^, ^24^] we further investigate several thiazolium salts and benchmarked them against the earlier literature reports. As a starting point of our investigations, commercially available thiazolium salts were used as catalysts in the catalytic self‐addition of acetaldehyde to form 3‐hydroxy‐2‐butanone (acetoin) as shown in Figure 1. Based on literature, Na_2_CO_3_ was used as a mild base to activate several pre‐catalyst (**NHC1‐8**) and yield the free carbene, *i. e*. active catalyst for the C−C bond formation reaction.[^23^, ^24^] To avoid run‐away of the acetaldehyde substrate during the reaction at high temperatures (>100 °C), reactions were carried out in sealed and pressure resistant glass reaction vessels inside a micro‐wave reactor. The results of these experiments are shown in Figure 1 **B**.

When using vitamin B1 or thiamine hydrochloride (**NHC1**) as pre‐catalyst in neat acetaldehyde, an acetoin yield of about 15 % was attained after 30 min of reaction with an exceptionally high selectivity towards the acetoin. Clearly, the mild base is sufficient in strength to activate the catalyst, but too weak to catalyse unwanted aldol‐side reactions, or the acetoin formation has a much higher reaction rate compared to the aldol reaction. Interestingly, when using the double amount of base (2.3 mol%) no improvement to the described result was obtained, although it is amendable that there is some base needed to deprotonate the thiamine hydrochloride prior to generating the catalytically active carbene *via* a second deprotonation. With 5‐(2‐hydroxyethyl)‐3,4‐dimethylthiazol‐3‐ium iodide (**NHC2**) as pre‐catalyst, acetoin was obtained in 28 % yield. To further investigate the role of the side‐group attached to the nitrogen atom, steric hindrance was increased by introducing an ethyl‐ (**NHC3**) and a benzyl‐group (**NHC4**, **NHC5**), respectively. Increasing the steric hindrance of the substituent on the quaternary carbon atom results in an increase in catalytic activity. For **NHC3** around 80 % acetoin yield is obtained. For **NHC4** and **NHC5** nearly quantitative (>99 %) yield, at *S* > 99 % is obtained after 30 minutes of reaction time. In order to identify the most active catalyst in the reaction, the experiments with **NHC4** and **NHC5** were repeated using a shorter reaction time of only 15 minutes. Both catalysts yield very comparable results of 51 % and 59 % acetoin yield respectively at a very high selectivity *S* >99 %. The results obtained here also hint, that the substituent on the quaternary nitrogen atom has a significant influence on the catalytic performance, whereas the influence of the substituent on the [5] position of the thiazole ring has much less of an influence (**NHC4** and **NHC5**).

Strikingly, even further increasing the steric hindrance of the side‐group by changing the benzyl for a mesityl group did not beneficially influence the catalytic performance of the system. With **NHC6**, 76 % product yield was attained. Apart from thiazolium salts as pre‐catalyst, an imidazolinium‐ and imidazole‐based compound (**NHC7** and **NHC8**, respectively) were tested as pre‐catalyst. Both respective carbenes are persistent once activated due to the mesomeric effect of the mesityl groups.^[27]^ Interestingly, imidazolinium‐based **NHC7** showed catalytic activity and 92 % acetoin could be obtained after the reaction, whereas imidazole‐based **NHC8** did not lead to an observable product formation (<1%). These findings hint that the delocalisation of the electrons in the NHC ring also has an influence on the productivity of the system.

**Scheme 2:**
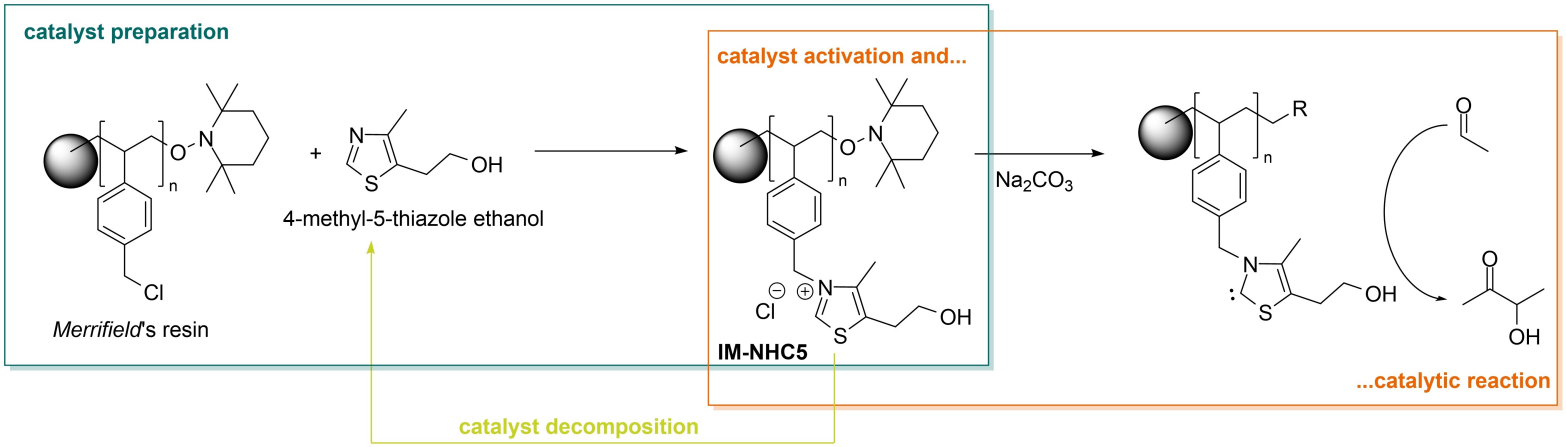
Overview of the preparation of **IM‐NHC5** *via* covalent linking of 4‐methyl‐5‐thiazole ethanol onto *Merrifield′s* polymer resin as well as the *in‐situ* activation reaction with Na_2_CO_3_ to yield the catalytically active immobilised carbene species as well as a possible catalyst degradation pathway, known as the reverse *Menshutkin* reaction.[^34^, ^35^, ^36^, ^37^]

In order to intensify the process, more experiments were carried out with the best performing carbenes from the above series (i. e. **NHC4**, **NHC5**, and **NHC7**). No notable recycling strategies like extraction of the resulting products showed any promising results (see Table S1 to Table S3 in the supporting information). Also, distillation remained fruitless, possibly owing to the instability of the active carbene at high temperatures. Instead, we turned our attention to an often‐applied technique to facilitate catalyst recycling: heterogenization by immobilization of the catalyst.[^28^, ^29^, ^30^, ^31^] More specifically, covalent linking of a thiazole compound onto an organic polymer resin (*Merrifield*’s peptide resin) was used as immobilization strategy.[^32^, ^33^] As this strategy is in principle possible with both **NHC4** and **NHC5**, we chose to reside with the immobilization of **NHC5** due to the cheap and readily available corresponding thiazole compound, 4‐methyl‐5‐thiaozle ethanol. Furthermore, since **NHC5** was regarded as more stable, the choice was made for this catalyst. Similar to the homogeneous pre‐catalysts, the heterogeneous pre‐catalyst can be activated by a base to *in‐situ* generate the active carbene catalyst.[^24^, ^30^, ^32^, ^33^] Commercially available chloro‐methyl polystyrene (~5.5 mmol ⋅ g^−1^ Cl purchased from *Sigma*) was reacted with 4‐methyl‐5‐thiazole ethanol to afford the immobilised pre‐catalyst **IM‐NHC5** which structurally resembles **NHC5** (Scheme 2). Due to the nature of the support, the final catalyst is obtained as small polystyrene beads which are easily separable after the reaction.

**Figure 2:**
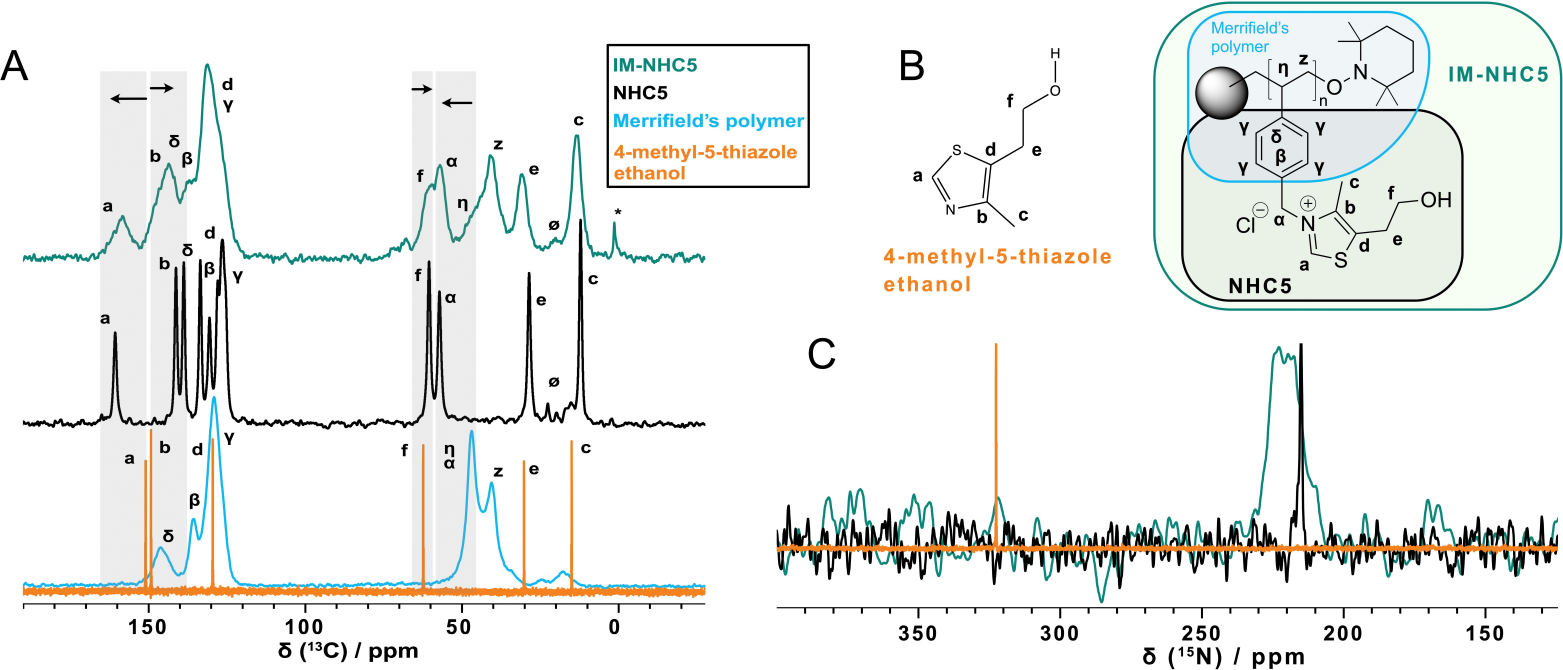
**A**: ^1^H‐^13^C CP spectra of *Merrifield*’s peptide resin (cyan spectrum), **NHC5** (black spectrum) and **IM‐NHC5** (green spectrum). In case of 4‐methyl‐5‐thiazole, a direct‐pulsed ^13^C spectrum is shown (orange spectrum). **B**: The atom labelling used plotted on the chemical structure. **C**: ^15^N NMR spectra of a 4‐methyl‐5‐thiazole ethanol solution, as well as solid **NHC5** and **IM‐NHC5**. For 4‐methyl‐5‐thiazole, ^1^H‐^15^N INEPT (orange line) and for **NHC5** (black line) and **IM‐NHC5** (green line) ^1^H‐^15^N CP spectra have been recorded. The spectra have been collected at 11.7 T and 14.0 kHz MAS. Spectra are scaled to the intensity of the 130 ppm (^13^C) and 220 ppm (^15^N) resonances. * denotes a resonance from a silicone sample spacer used. Ø denotes MAS sidebands ^13^C resonances experiencing significant chemical‐shift changes upon catalyst immobilization are highlighted by grey rectangles in **A**.

The successful formation of the **IM‐NHC5** catalyst was supported by solid‐state NMR spectroscopy. In that vein, ^13^C‐detected solid‐state NMR experiments have been performed on **IM‐NHC5**, **NHC5**, *Merrifield*’s polymer resin and 4‐methyl‐5‐thiazole ethanol (Figure 2 **A**, for the chemical structures see Figure 2 **B**). While **IM‐NHC5** and **NHC5** are both solid entities for which ^1^H‐^13^C cross‐polarization (CP) experiments have been collected, 4‐methyl‐5‐thiazole ethanol is liquid at ambient conditions and thus a direct‐pulsed ^13^C spectrum has been recorded. Comparison of the ^1^H‐^13^C CP spectra reveal that the spectrum of **IM‐NHC5** resembles the individual spectra of **NHC5** and *Merrifield*’s peptide resin confirming successful catalyst immobilization. Particularly the comparison of the spectrum of **IM‐NHC5** with the^13^C spectrum of 4‐methyl‐5‐thiazole ethanol reveals some pronounced chemical‐shift changes (such as the carbon nuclei of the thiazole ring, labelled with a and b in Figure 2) upon catalyst immobilization which are highlighted in Figure 2 **A**. The change in the ^13^C chemical‐shift values of site “a” and “b” by around±10 ppm is most likely caused by covalent bonding between the polymer and the 4‐methyl‐5‐thiazole ethanol instead of an unspecific adsorption on the polymer surface. Broadening in the resonances of **IM‐NHC5** are related to structural disorder in the polymer.

In addition, ^15^N‐detected solid‐state NMR spectra were recorded for the immobilized catalyst **IM‐NHC5**. For comparative reasons, ^15^N‐detected solid‐state NMR spectra of the catalyst precursor **NHC5** and 4‐methyl‐5‐thiazole ethanol, were recorded as well (Figure 2 **C**). Again, CP experiments were collected for **IM‐NHC5** and **NHC5**, while for 4‐methyl‐5‐thiazole ethanol a ^1^H‐^15^N insensitive nuclei enhanced by polarization transfer (INEPT) experiment has been performed. Although ^15^N‐detected solid‐state NMR experiments suffer from low sensitivity, thus long experimental measurement time is required (around 25 to 78 h in this case), the ^15^N chemical‐shift value provides important insights into the success of the immobilization reaction. The thiazole nitrogen nucleus of 4‐methyl‐5‐thiazole ethanol resonates at around 322 ppm (orange spectrum in Figure 2 **C**, referenced with NH_4_Cl at 39.3 ppm relative to liquid NH_3_ ^[38]^, and experiences a significant low‐frequency shift of around 108 ppm in **IM‐NHC5** upon covalent‐bond formation with Merrifield′s polymer (green spectrum in Figure 2 **C**). Shielding effects of similar magnitude in ^15^N NMR spectra have been reported for sp^2^ hybridized nitrogen atoms upon protonation.^[39]^ The observation of such a large ^15^N chemical‐shift difference upon covalent bond formation has been further supported by density functional theory (DFT) calculations of the ^15^N magnetic‐shielding tensors for 4‐methyl‐5‐thiazole ethanol and **NHC5** molecules, which resulted in an absolute value difference between the isotropic ^15^N chemical‐shift values of |Δδ(^15^N)|=122.9 ppm for these two compounds (for further details see SI), which is of similar magnitude than the experimentally observed low‐frequency shift. The ^15^N resonance of **IM‐NHC5** is significantly broadened in agreement with chemical‐shift distribution effects often observed in polymeric species. The ^15^N resonance of the thiazole nitrogen nucleus in **NHC5** is detected at 215 ppm and thus at a similar chemical‐shift value than for the **IM‐NHC5** further supporting the successful grafting of the polymer with 4‐methyl‐5‐thiazole ethanol to afford the grafted pre‐catalyst.

**Figure 3:**
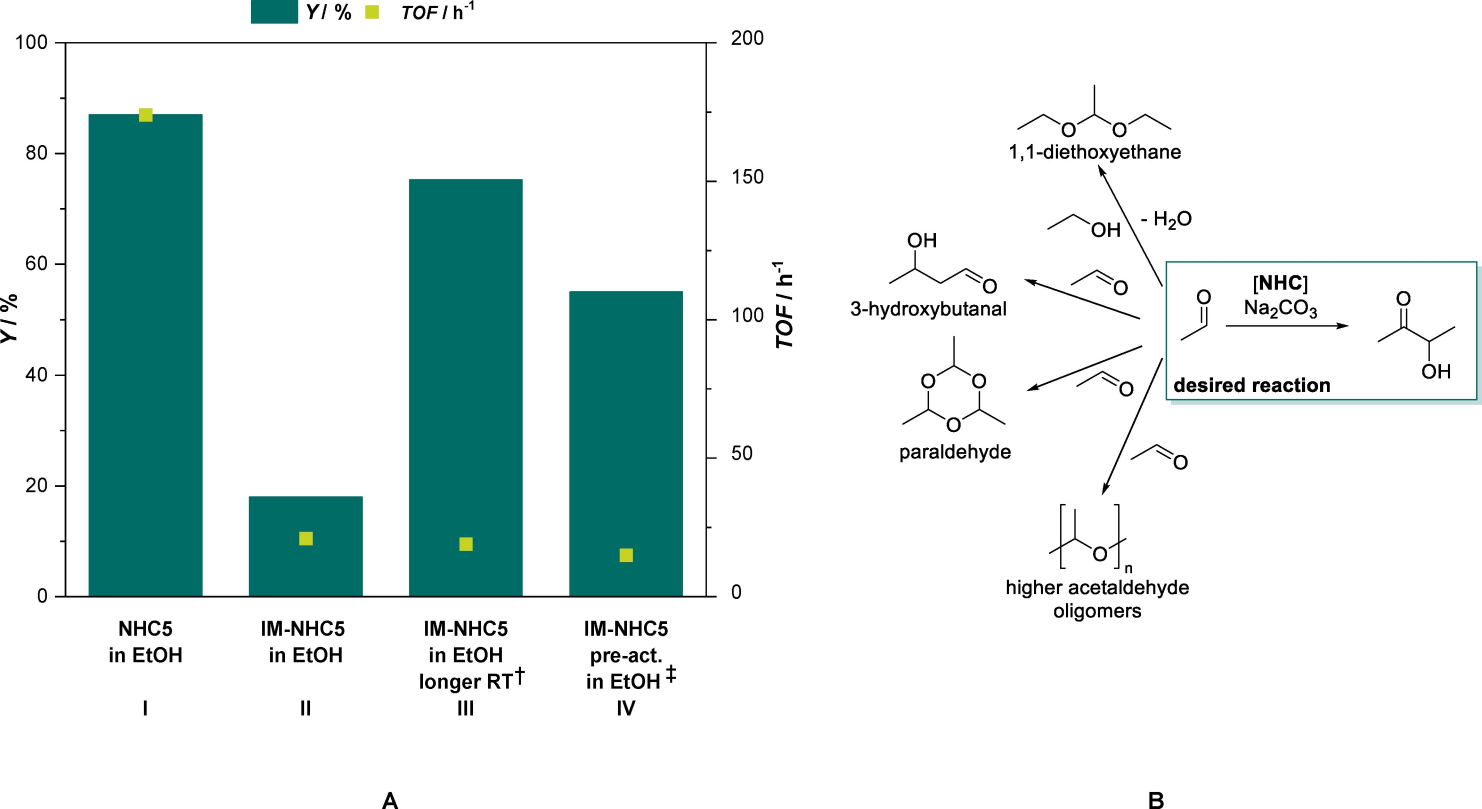
**A**: Catalytic results from various experiments using either **NHC5** or **IM‐NHC5** as a catalyst in the acetaldehyde self‐addition. **B**: an overview of side products that were obseved in minor quantities throughout the experiments. Reaction conditions: *V*(acetaldehyde)=1.0 mL, *n*(acetaldehyde)=18 mmol, *c*(cat)=1.0 mol%, *c*(Na_2_CO_3_)=1.3 mol%, *V*(EtOH)=5.0 mL, *T*=100 °C, *t*=0.5 h. † *c*(**IM‐NHC**)=1.0 mol%, *c*(Na_2_CO_3_)=1.3 mol%, *V*(EtOH)=1.0 mL, *T*=70 °C, *t*=2 h. ≠ pre‐activated using equimolar amount of KO^*t*^Bu before adding substrate to avoid base catalysed side reactions (aldol) at otherwise identical reaction conditions to †.

After successfully proving the immobilization by covalent linking, we subjected the **IM‐NHC5** catalyst to extensive testing to ‘standard’ reaction conditions, *i. e*. neat, 100 °C, 30 min, 1 mol% catalyst and 1.3 mol% base and *in‐situ* activation of the catalyst (*vide supra*). Unfortunately, this first experiment with **IM‐NHC5** in neat conditions only led to a very low acetoin yield of <1%. However, in a coupled ethanol‐to‐acetoin upgrading sequence, tolerance towards residual ethanol in the acetaldehyde‐feed stream was already illustrated.^[24]^ In our work, we were even able to show that ethanol can also be used as solvent for the **NHC5**/Na_2_CO_3_ ‐system, yielding 87 % acetoin with very high selectivity >98 % (Figure 3 **A**, experiment **I**). We subjected the solid **IM‐NHC5** catalyst to the same solvated reaction conditions and found that after 30 min of reaction time, about 18 % of acetoin could be obtained (Figure 3 **A**, experiment **II**). As main by‐product 1,1‐diethoxyethane as well as paraldehyde were observed from GC‐MS measurements, resulting in an overall selectivity of *S*=82 % (Figure S4 in the SI). Interestingly, these side‐products could not be identified from neat reactions discussed earlier. An overview of the possible side‐products observed in the reaction system, is given in Figure 3 **B**.

Experiment **II** was repeated for 2, 8 and 16 hours at 70 °C (to suppress assumed decomposition of the **IM‐NHC5** catalyst for these longer reaction times). After 2 h, an almost four‐fold increase in yield to 75 % was obtained (**III**, Figure 3 **A**). For 8 and 16 h of reaction time, nearly quantitative amounts of acetoin were obtained (>95 %) at an excellent selectivity >96 % throughout the experiments (Table S4 in the SI). Again, for these experiments, 1,1‐diethyoxyethane was observed as main by‐product. Using ethanol as solvent, thereby decreasing the partial concentration of acetaldehyde in solution leads to the (base‐)catalysed by‐product formation. Evidence for this assumption was given by a blind‐experiment where pristine PS beads and Na_2_CO_3_ was reacted with acetaldehyde in ethanol (Table 1, entry 1.1). For this experiment, 1,1‐diethoxyethane as well as some 3‐hydroxybutanal (aldol‐product) was observed (Figure 3 **B**). From GC‐FID measurements, some higher acetaldehyde‐oligomers could be observed as well. For this blind experiment in absence of catalyst, conversions of acetaldehyde were found to be very low, resulting in negligible amounts of acetaldehyde oligomers.

**Table 1:** Results of the verification experiments using different combinations of catalytic systems in the acetaldehyde self‐addition as well as the hot‐filtration‐test carried out with the **IM‐NHC5**/Na_2_CO_3_ catalytic system.^[a]^

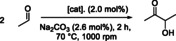				
Entry	Catalytic system	*X*(acetaldehyde)^[b]^ (%)	*Y*(acetoin)^[b,c]^ (%)	∑*Y*(side‐products)^[b,d]^ (%)
1.1	PS beads/Na_2_CO_3_	2.5	≪0.1	2.5
1.2	4‐methyl‐5‐thiazole ethanol/Na_2_CO_3_	2.6	≪0.1	2.6
1.3^[e]^	–	2.9	≪0.1	2.9
1.4^[f]^	**IM‐NHC5**/Na_2_CO_3_	38	34	4
1.5^[g]^	entry 1.4 after *hot filtration test*	41	35	6

[a] reaction conditions: *V*(acetaldehyde)=1.0 mL, *n*(acetaldehyde)=17.8 mmol, *V*(EtOH)=1.0 mL, *c*(cat.)=2 mol%, *c*(base)=2.6 mol%, *T*=70 °C, *t*=2 h, 1000 rpm. [b] As determined by GC‐FID using mesitylene as internal standard. [c] Acetoin yields including the yield of dimeric acetoin *R*_t_=11.1–11.4 min. [d] side products include 1,1‐diethoxyethane, paraldehyde and others as shown in Figure 3**B**. [e] in absence of catalyst/base. [f] *c*(**IM‐NHC**)=1.0 mol%, *c*(Na_2_CO_3_)=1.3 mol%, *V*(EtOH)=1.0 mL, *T*=70 °C, *t*=2 h, 1000 rpm. [g] after removal of the **IM‐NHC5**/Na_2_CO_3_ catalyst system *via* cannular filtration, the supernatant solution was reacted for another *t*=2 h, *T*=70 °C.

So far, only results were discussed where a mild inorganic base (Na_2_CO_3_) was used to activate the pre‐catalyst and yield the active carbene species. Although Na_2_CO_3_ is sufficient to activate **IM‐NHC5**, using solid base might cause problems in future process intensification steps. On the other hand, experiments with stronger organic bases such as NaOEt and KO^*t*^Bu caused immediate and vigorous polymerisation of the reactive acetaldehyde. In order to try and attempt to avoid the use of solid inorganic base, we turned our attention to the use of diethylamine (Et_2_NH) as weak organic base which is dissolvable in ethanol. However, with Et_2_NH the formation of 1,1‐diethoxyethane, 3‐hydroxybutanal and other higher oligomers of acetaldehyde became favourable over the acetoin formation (Table S5, in the SI). Apparently, Et_2_NH is not sufficient enough to deprotonate the solid carbene pre‐catalyst and yield the active carbene. This has also been illustrated by *Wang* and *Chen* in the catalytic self‐addition of 5‐HMF to C_10_ furoins.^[32]^ Therefore, we turned to a pre‐activation sequence where different strong organic bases were reacted with the **IM‐NHC5** catalyst before adding acetaldehyde substrate. With this approach, 55 % acetoin yield was obtained after pre‐activating the catalyst with KO^*t*^Bu in ethanol before removing the base‐containing pre‐activation solution and adding acetaldehyde substrate (Figure 3 **A**, experiment **IV**). In these pre‐activating experiments, 1,1‐diethoxyethane and other aldol products were found to be below detection limits of GC‐FID. Although the possibility of pre‐activating the **IM‐NHC5** seems promising for continuous flow applications, we decided to further investigate the **IM‐NHC5**/Na_2_CO_3_ system due to its outstanding productivity, selectivity and exceptional ease of use.

### Batch Recycling with IM‐NHC5/Na_2_CO_3_

**Figure 4:**
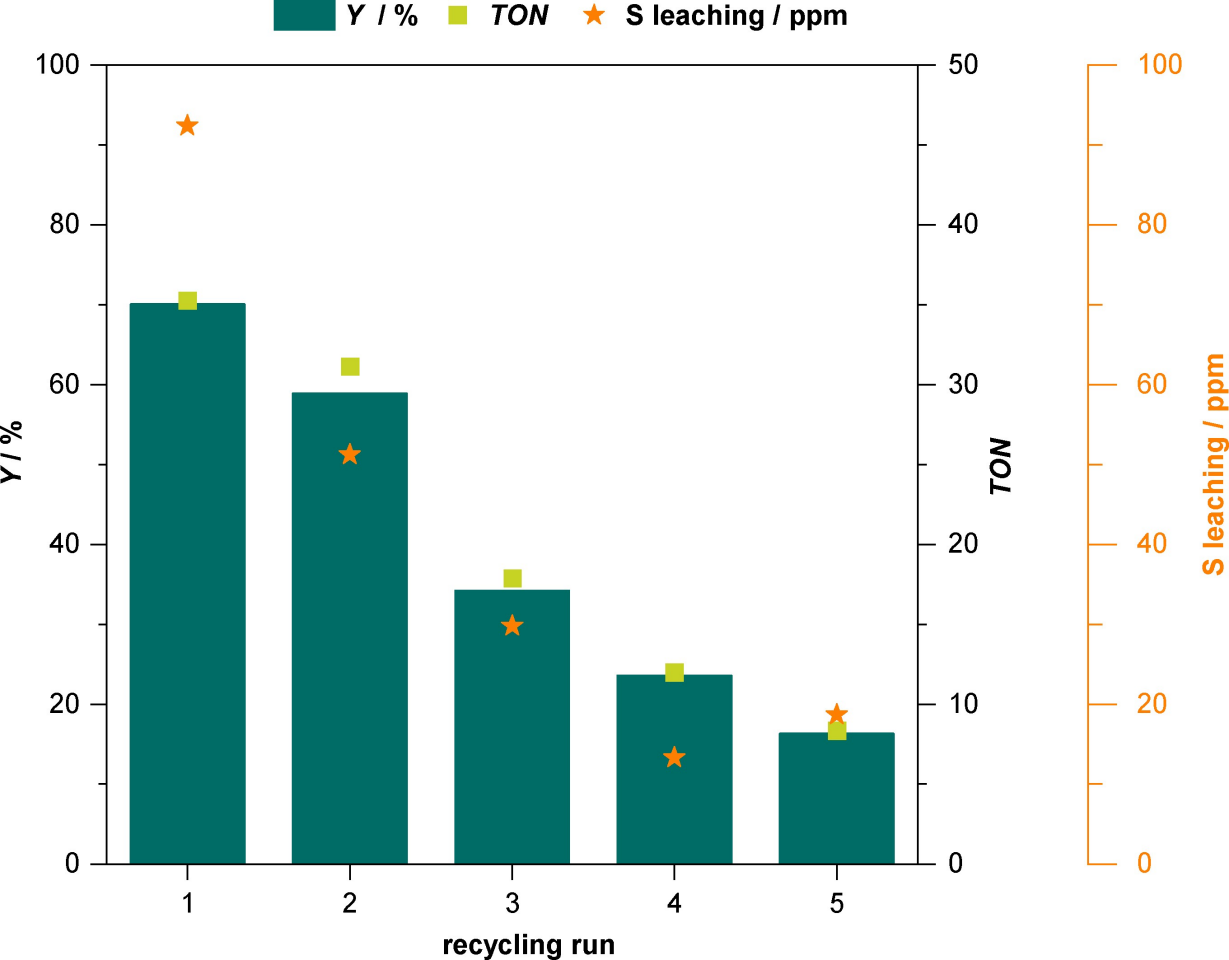
Catalytic results from five consecutive batch recycling runs using the **IM‐NHC5**/Na_2_CO_3_ catalytic system in the acetaldehyde self‐addition. Reaction conditions: *V*(Stock)=2.0 mL for each run, *m*(**IM‐NHC5**)=119.8 mg (0.4 mmol, 2.0 mol%), *m*(Na_2_CO_3_)=53.4 mg (0.5 mmol, 2.6 mol%), *T*=70 °C, *t*=2 h, 1000 rpm. Yields determined by GC‐FID using mesitylene as internal standard. For all runs, mass‐balances Σ*Y*>92 % were obtained.

We studied batch recycling with the reaction conditions of the best‐performing **IM‐NHC5**/Na_2_CO_3_‐system, 2 mol% catalyst, 2.6 mol% of base, 70 °C, and 2 h of reaction time. The results of the five consecutive batch recycling experiments are visualised below in Figure 4.

After each run, the post‐reaction solution was retrieved from the reaction vessel using cannular filtration while the catalyst beads stayed in the reactor. New substrate containing stock‐solution was then added. Hence, no intermittent catalyst re‐activation procedure was carried out. Care was taken to keep the active catalyst in protected Ar environment during filtration of post‐reaction solution and during the addition of fresh substrate. Due to the nature of the **IM‐NHC5** beads no solid NHC catalyst was lost. Due to the limited solubility of Na_2_CO_3_ in ethanol, the amount of base could be kept constant as well. This was further proven by XRF analysis after each run. No elevated levels for Na were detected from the post‐reaction samples (<17 ppm), hinting that the amount of base was kept constant. In the first catalytic run, a yield of 72 % acetoin is attained (Figure 4). Interestingly, already from the first recycling run, elevated sulphur concentrations were found in the post‐reaction solution. For the second recycling run, a clear decrease in catalytic activity can be observed as around 60 % acetoin is obtained. Over the course of three successive runs, the catalytic activity decays further, yielding 16 % of acetoin in the fifth run. As shown in Figure 4, after every run, an elevated level of sulphur is found to be present in the post‐reaction solution. As can be seen from Scheme 2 every catalytically active centre in **IM‐NHC5** contains a single sulphur atom. Presence of sulphur in the post‐reaction solution therefore can be regarded as indication that the catalyst somehow decomposes. To exclude that this apparent decomposition stems from the handling of the batch recycling experiments, we switched from a batch recycling to a continuous flow application using the **IM‐NHC5**/Na_2_CO_3_ system.

### Continuous Flow Applications of the IM‐NHC5/Na_2_CO_3_ System

**Figure 5:**
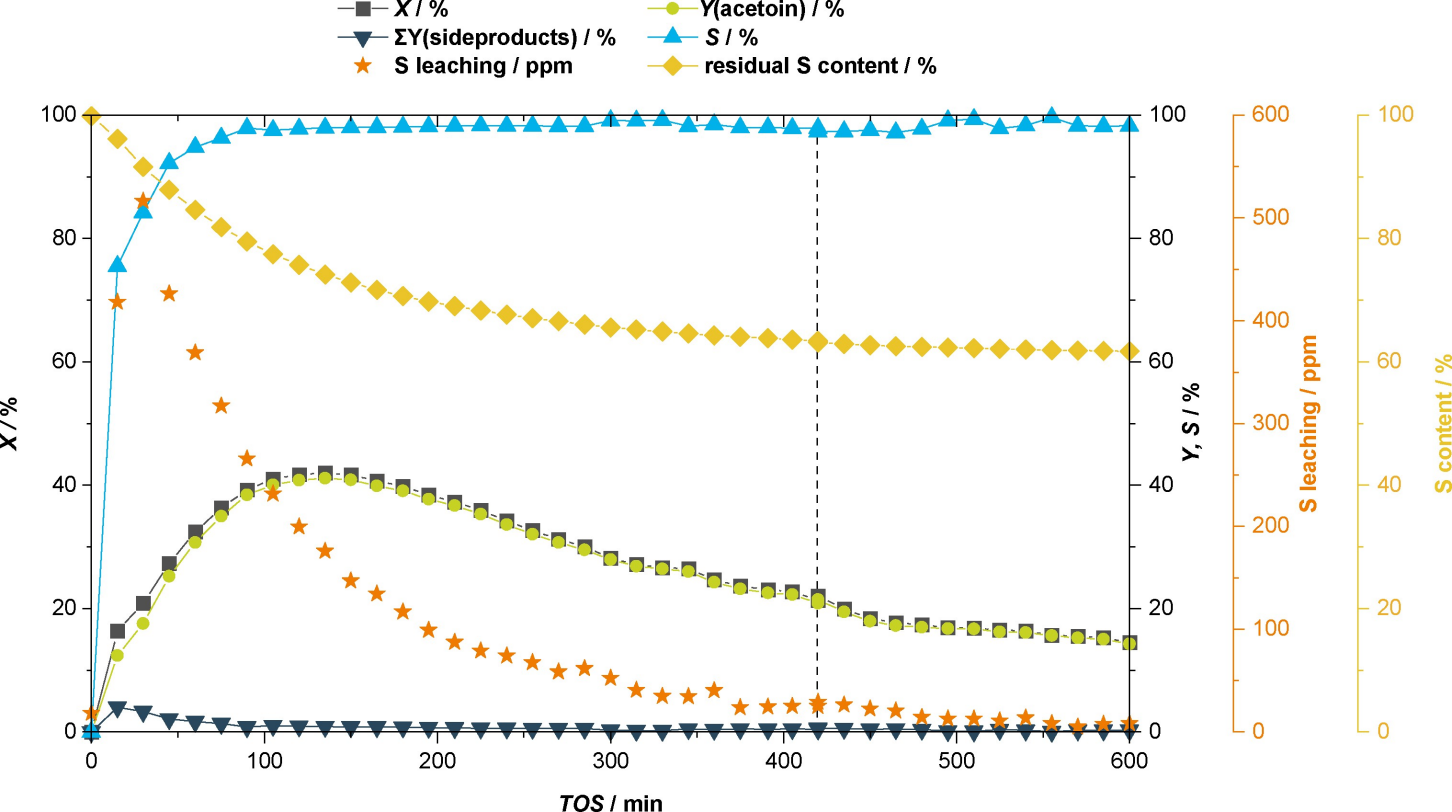
Results of the continuous flow acetaldehyde self‐addition in a CSTR setup over a total of 10 h TOS. Graph shows the conversion *X*, yield *Y* and selectivity *S* towards acetoin over TOS as well as the calculated residual Sulphur content (%) of the catalyst and the measured leaching of S (ppm) for samples taken every 15 minutes for a duration of 4 minutes. Reaction conditions: stock‐solution containing acetaldehyde (250 mL, 194.7 g, 4.4 mol), EtOH (250 mL, 196.4 g, 4.5 mol) and mesitylene (29.0 mL, 24.86 g), *V̇* (stock)=0.475 mL ⋅ min^−1^, *m*(**IM‐NHC5**)=1.7386 g (3.24 mmol ⋅ g^‐1^, 5.63 mmol NHC, 2 mol%), *m*(Na_2_CO_3_)=775.9 mg (7.32 mmol, 2.6 mol%), *T*=70 °C, *τ*=2 h, 1000 rpm. Mass‐balances Σ*Y* >90 % were obtained. Dotted line at *t*=420 min TOS, indicates that the reaction was terminated over‐night and re‐started the next day.

For the relevant CSTR experiments that will be discussed in the following, a residence time of *τ*=2 h was chosen to mimic the individual batch recycling experiments. One objective of these experiments is to further study and understand the leaching behaviour by tracing the amount of sulphur in the post‐reaction solution. Furthermore, we were intrigued to see if operating the system in continuous flow leads to an enhancement in the overall process *i. e*. improve the stability of the catalytic system and achieve higher TONs. Conversion, yield, selectivity and the leaching‐over‐time behaviour of the first continuous flow experiment carried out, are shown below in Figure 5.

As it can be seen from the above graph, after a start‐up phase of approximately 120 min TOS, the maximum catalytic activity of the system is reached, resulting in 41 % acetoin yield at 42 % acetaldehyde conversion at a selectivity towards acetoin of >97 %. Compared to a typical batch experiment, the obtained yields/conversions are lower (Figure 4, *Y*(acetoin)=70 % after 2 h). The lower yield in the continuous flow experiment can be explained by the fact that the residence time is only an average. Within the continuous flow setup, channelling can occur, giving one possible explanation for runaway of acetaldehyde. Excellent selectivity values *S*>97 % are obtained throughout the duration of the experiment. Only within the first 2 h into the reaction, side‐products like paraldehyde, 1,1‐diethoxyethane and of 3‐hydroxybutanal (aldol product) were identified in GC‐FID with a combined yield <2% after 100 min TOS. After reaching a maximum in conversion and yield, the activity of the catalyst continuously drops to around 15 % conversion/yield after 600 min TOS. In addition to the conversion‐/yield‐ and selectivity‐time curves, the calculated residual sulphur content (%) as well as the sulphur leaching (ppm) is also shown in Figure 5. In order to construct the plot of residual sulphur content (*i. e*. residual active catalytic centres) the spent catalyst was analysed by ICP and the amount of sulphur of the catalyst was found to be 6.41 % or about 2.00 mmol ⋅ g^−1^ whereas the pristine **IM‐NHC5** has a loading of 3.24 mmol ⋅ g^−1^. Around 1.24 mmol ⋅ g^−1^ catalytically active components or 38 % of the initial amount is lost during the reaction. The leaching trend seems to be rather exponential in nature. Lots of leaching is observed in the beginning. From around 300 min TOS onward, a more or less constant leaching, hence constant loss in catalytic activity is observed towards the end of the reaction. Interestingly, terminating the reaction after 420 min TOS (*i. e*. quickly cooling to rt) and leaving the reactor over night before continuing the experiment on the next day, does have a significant influence on the system. Although conversions and yields steadily drop, the selectivity remains high, also after restarting the reaction the next day. Like pointed out earlier, the decrease in productivity of the system is caused by a loss of catalytically active component. Unfortunately, taking the system into a continuous operation mode, did not suppress leaching. Hence, the cause of catalyst decomposition must have an underlying molecular effect.

**Figure 6:**
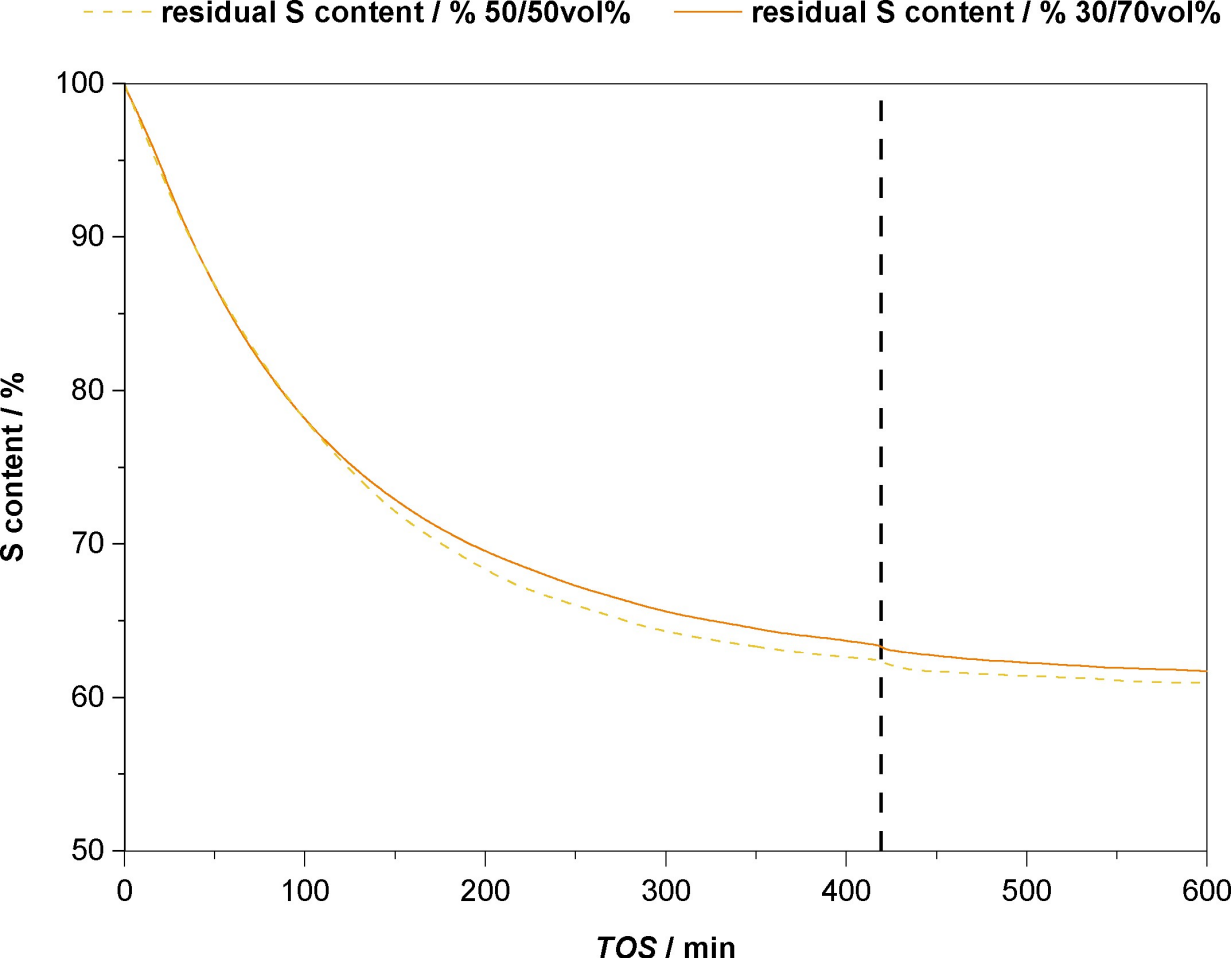
Comparison of the sulphur leaching‐over‐time behaviour of two continuous flow experiments using different concentrations of acetaldehyde containing stocksolution. Residual sulphur content was calculated based on the leaching of S (ppm) aquired from XRF measruements from outlet samples taken every 15 minutes for a duration of 4 minutes. Reaction conditions: stock‐solution containing acetaldehyde, *V̇*(stock)=0.475 mL ⋅ min^−1^, *m*(**IM‐NHC5**)=1.0445 g (3.24 mmol ⋅ g^−1^, 3.38 mmol NHC, 2 mol%), *m*(Na_2_CO_3_)=467.3 mg (4.49 mmol, 2.6 mol%), *T*=70 °C, 1000 rpm. Dotted vertical line at 420 min TOS, indicates that the reaction was terminated over‐night and re‐started the next day. The full conversion/yield over time data for the experiment using a 3 : 7 mixture of acetaldehyde in ethanol is given in the supporting information in Figure S5.

Decrease of catalytic activity over time has also been observed by *Lemaire et al*. when using thiazolidine‐based NHCs in the catalytic cleavage of vegetable oil derivatives bearing similar α‐hydroxyketone moieties.^[37]^ It was proposed that catalyst deactivation can occur from the reaction of the carbene species with a nucleophile. In our specific case, the formed acetoin *i. e*. the reaction product could be such weak nucleophile. One way of circumventing product induced catalyst decomposition, is to reduce the concentration of substrate and consequently of product in the reaction system. In order to verify the hypothesised product induced catalyst decomposition, another continuous flow experiment was carried out where instead of a 1 : 1 mixture (Figure 5) of acetaldehyde and ethanol a 3 : 7 mixture was used. The comparison of the leaching curves of this continuous flow experiment are graphically shown below in Figure 6. The full conversion, yield, and selectivity‐over‐time behaviour attained for this experiment is shown in SI as the trends in conversion, yield and selectivity are very similar to the ones observed for experiment carried out with the 1 : 1 acetaldehyde containing mixture discussed earlier.

Interestingly, when both plots of the residual sulphur content over time are compared, almost no difference can be observed. The observed leaching‐over‐time behaviour is very similar in both experiments. Based on ICP analysis, the spent catalyst of the experiment with a 1 : 1 mixture (Figure 5) had a residual loading of 2.00 mmol ⋅ g^−1^, the spent catalyst from the experiment with the 3 : 7 mixture had 1.97 mmol ⋅ g^−1^ catalytically active component left. Both experiments result in very similar catalyst leaching‐over‐time behaviour and similar loadings of the spent catalyst. This finding does not support the earlier formulated hypothesis that the product is able to decompose the catalyst. Carrying out a complete blind‐experiment without any acetaldehyde present in the stock‐solution, gave a residual loading of 2.24 mmol ⋅ g^−1^ (SI, Figure S6). The value obtained residual S loading after the blind experiment is not significantly different from the ones obtained earlier after either an experiment with a 1 : 1 or a 3 : 7 mixture. Hence, if product induced catalyst decomposition plays a role in the observed leaching behaviour, its effect is minimal.

**Figure 7:**
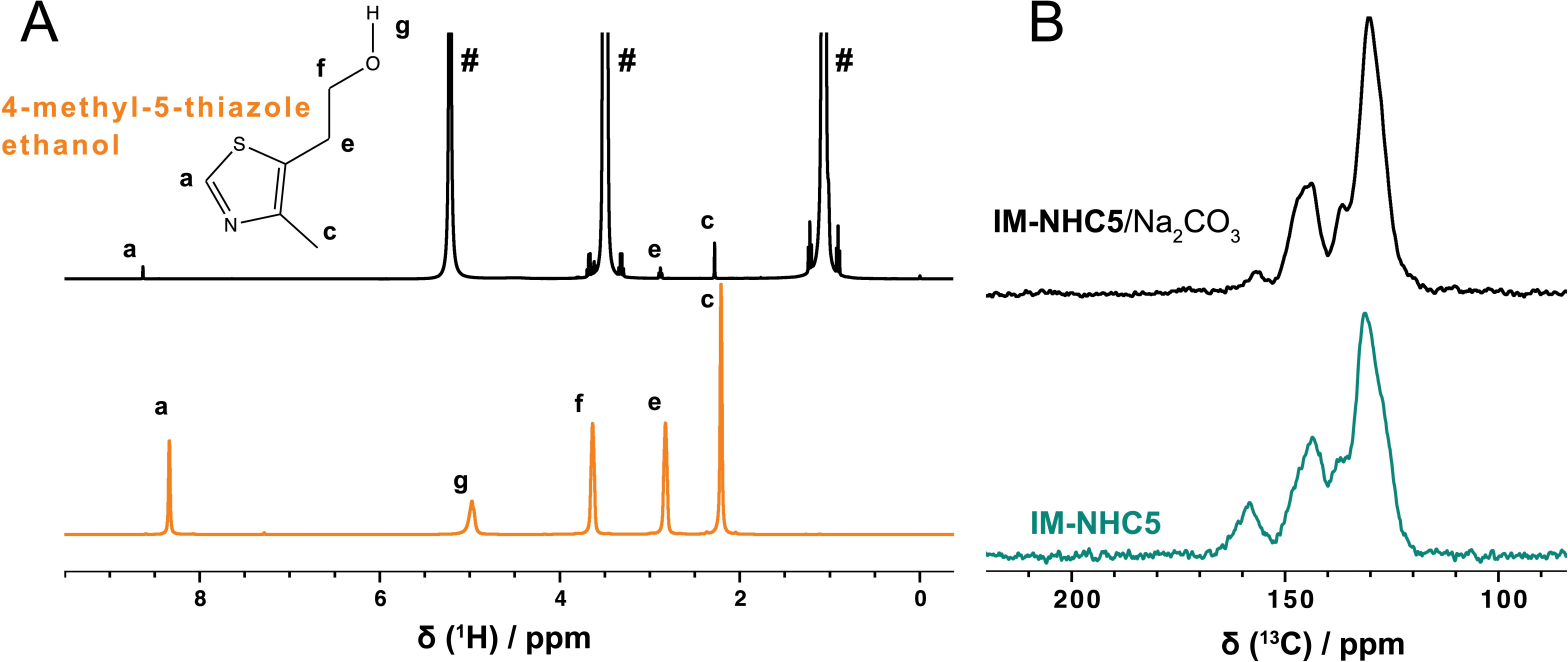
**A**: Comparison of ^1^H solution‐state NMR (400 MHz, EtOD‐*d*_6_, rt) spectrum of the postreaction of a blind‐experiment where **IM‐NHC5**/Na_2_CO_3_ was heated in EtOH for 2 h at 70 °C (top) as well as the pure component ^1^H solution‐state NMR (400 MHz, CDCl_3_, rt) spectrum of 4‐methyl‐5‐thiazole ethanol (bottom) including the resonance assignments. **B** ^1^H‐^13^C CP NMR spectra of **IM‐NHC5**/Na_2_CO_3_ (black) and IM‐NHC5 (from Figure 2 **A**, green). Assignments are shown above in Figure 2 **A** and 2B (for the labelling used in B see Figure 2). The spectra were recorded at 11.7 T and an MAS frequency of 14.0 kHz. # denote ^1^H peaks of the solvent ethanol that are also cut from above.

To further understand the leaching of the catalyst and to disclose a possible decomposition pathway, we resided once more to NMR studies to obtain a more profound knowledge on the leaching of sulphur. As starting point of these investigations, an experiment was carried out with the **IM‐NHC5**/Na_2_CO_3_ catalyst system in absence of any substrate at otherwise identical reaction conditions. After the reaction, the post‐reaction content was analysed using ESI‐MS, ^1^H‐solution‐state NMR spectroscopy as well as XRF. Again, for the XRF about 320 ppm S was found whereas Na levels were below detection limits (<17 ppm). As far as the ESI‐MS is concerned, the most prominent peak at *m/z*=144.1 was found from the positive scan which is a strong indication for the presence of 4‐methyl‐5‐thiazole ethanol which has a mass of 143.21 g ⋅ mol^−1^ (SI, Figure S3). From the ^1^H‐NMR spectrum (400 MHz, EtOD‐*d*_6_, rt) on the post‐reaction content signals were observed at δ=8.62 (s, 1H), 2.88 (t, *J*=6.7 Hz, 2H), 2.28 (s, 3H) ppm. Comparison with the pure component spectrum of 4‐methyl‐5‐thiazole ethanol and literature data strengthens the assumption that 4‐methyl‐5‐thiazole ethanol is present in the post‐reaction solution (Figure 7). A zoom into the aromatic region of the solid‐state NMR ^13^C CP‐MAS spectrum of the catalyst after the blind experiment in absence of substrate is shown in Figure 7 **B** and indeed also reveals decreasing resonances (e. g. the resonance at 160 ppm, the full spectra are given in the SI in Figure S9) assigned to the immobilized catalyst supporting the release of the catalyst during the reaction.

Apart from these signals, no other possible (dissolved) decomposition product could be identified. Therefore, the most possible pathway of decomposition for **IM‐NHC** is the degradation yielding pristine resin and 4‐methyl‐5‐thiazole ethanol (Scheme 2). This degradation pathway, known as the reverse *Menshutkin* is well established in literature for imidazolium as well as thiazolium halide salts.[^34^, ^35^, ^36^, ^37^]

To verify that the leached compound and/or pristine resin has no residual catalytic activity, more blind experiments as well as a so‐called hot filtration test was carried out (Table 1).

As elucidated earlier, the catalytic activity of pristine support beads in combination with Na_2_CO_3_ is negligible. The same holds true for the combination of 4‐methyl‐5‐thiazole ethanol and Na_2_CO_3_ (entry 1.2). This is not surprising since the 4‐methyl‐5‐thiazole cannot be converted into an active carbene species. Both experiments show that upon decomposition of **IM‐NHC5** into pristine *Merrifield*’s resin as well as 4‐methyl‐5‐thiazole ethanol, hardly any residual catalytic activity of the system is to be expected. In absence of any catalyst or base, heating acetaldehyde and solvent (EtOH) at otherwise identical reaction conditions, did not lead to observable quantities of acetoin and mostly (aldol) by‐products were obtained at a low conversion of 2.9 % (entry 1.3).

In order to prove if the acetaldehyde addition proceeds indeed *via* the heterogeneous **IM‐NHC5** catalyst or some leached species of the catalyst into solution (*i. e*. homogeneous) a hot filtration test was carried out. After 1 h of reaction time, 34 % acetoin yield was obtained from GC‐FID (entry 1.4). The filtrate was continued to react for an additional hour but the reaction more or less ceased (entry 1.5). Interestingly, more side‐products were obtained after the filtration test, although the filtrate was absolutely clear and no residual base was present in the system as confirmed by XRF measurements. These results indicate that the reaction proceeds indeed *via* the heterogeneous carbene catalyst and that possible leached species do not exhibit catalytic activity in the acetaldehyde self‐addition.

## Conclusions

To the best of our knowledge, we are the first to successfully demonstrate the self‐addition of acetaldehyde in ethanol in both batch and continuous operation mode. The developed system was able to achieve a total turn‐over‐number of 111 and a space‐time‐yield of 0.13 kg ⋅ l^−1^ ⋅ h^−1^ at an average yield of 26 % acetoin through single‐pass‐conversion. A very active catalyst was found in homogeneous batch screening experiments and immobilized *via* grafting on *Merrifield* polymer resin. Extensive solid‐state NMR characterisation was applied. Based on the observations in ^13^C and ^15^N solid‐state NMR spectra, clear indication was found for the covalent linking between the catalyst and the polymer resin.

The catalyst system discussed herein, showed decomposition of the active species *via* the reverse *Menshutkin* reaction with increasing time‐on‐stream. During a typical 10 h run, 39 % of the catalyst decomposed and leached into solution as 4‐methyl‐5‐thiazole ethanol which was tracked by XRF and ^1^H‐solution‐state NMR analysis of the reactor effluent. Solid‐state NMR studies and comparison of pre‐ and post‐reaction catalyst measurements reveal decreasing resonances in the aromatic region of the ^1^H‐^13^C CP NMR spectra, supporting the decomposition of the catalyst during the reaction.

Future investigations with regard to the optimisation of the performance of the heterogeneous catalyst will focus on structural improvements made to the catalyst in order to supress the reverse *Menshutkin* reaction. It is amendable that mitigating this decomposition reaction can increase the stability and the overall productivity of the catalyst significantly. These structural improvements could include but are not limited to the change of the nucleophilic Cl‐ion by for example a less nucleophilic anion.

## Experimental Section

### General Considerations

EtOH, HPLC Gradient Rotisolv® (<0.1 % H_2_O) was purchased from *CarlRoth* and was used without further drying. Anhydrous acetaldehyde (>99.99 %) was purchased from *Sigma* and used without further purification. Mesitylene purchased from *Sigma* (98 %), used as internal standard for GC‐FID measurements, was dried over MgSO_4_ (obtained from *Sigma*) overnight. (Chloromethyl) polystyrene (*Merrifield*’s peptide resin, porous, extent of labeling: ~5.5 mmol/g Cl loading) as well as 4‐methyl‐5‐thiazole ethnanol (98 %) were also obtained from *Sigma*. 4‐methyl‐5‐thiazole ethanol was degassed and stored over molecular sieves (4 Å). For reactions carried out in inert gas atmosphere, argon supplied by AirLiquide (99.9999 purtiy) was used. Air sensitive chemicals and self‐made catalysts were stored in an Ar filled glovebox and standard Schlenk techniques were applied.

GC measurements were carried out on a Shimadzu Nexus GC‐2030 instrument equipped with a Restek Rtx‐1701 (30 m) column and flame ionisation detector (FID). Quantitative analysis was carried out by correlating the corrected signal intensity to the known amount of standard. Correction factors for acetaldehyde and acetoin were determined by calibrating a stock‐solution with known concentration. For the non‐calibrated side‐products, the *Sternberg* method was used to approximate the correction factors.^[40]^ Side‐product were identified using GCMS‐QP2020 purchased from Shimadzu equipped with the same Restek Rtx‐1701 (30 m) column.

ICP analysis was carried out by *Mikroanalytisches Labor Kolbe*. The samples were analysed for carbon, sulphur, nitrogen, hydrogen, oxygen and chloride content.

XRF analysis was carried using a *Spectro Xepos C* instrument with a Prolene® 12 μm foil for a measuring time of 600 seconds in a mixed He/Air atmosphere at an Energy‐level (*E*) of 3 keV–19 keV. Each energy range was measured for 150 seconds. The energy ranges were divided as follows: 6 keV≤*E*≤19 keV; *E*>19 keV, 3 keV≤*E*≤6 keV and *E*<3 keV. For a typical measurement, 1.0 mL of sample was filled into a cuvette with a diameter of 20 mm and an inner diameter of 16 mm. The bottom of the cuvette is made of a 12 μm thick Prolene® foil. The sample thickness was approximately 6 mm. The device is recalibrated once a day. For this purpose, a glass tablet with a certain composition of elements is used for referencing.

### Preparation of IM‐NHC5


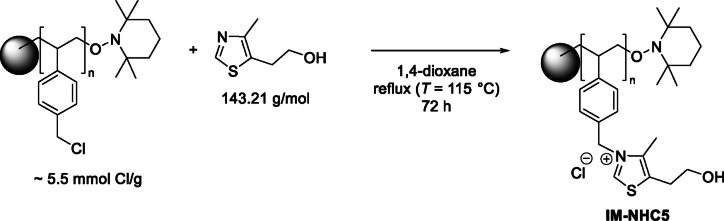


Experiments described within this work, were carried out using the same batch of **IM‐NHC5** which was prepared as follows: To an overn‐dried 500 mL *Schlenk* flask was added (chloromethyl) polystyrene (10 g, ~55.5 mmol Cl, 1.0 eq), 1.4‐dioxane (200 mL) as well as 4‐methyl‐5‐thiazole ethanol (19.987 g, 138.7 mmol, 2.5 eq). The mixture was stirred under reflux in an Ar atmosphere. After 72 h, the reaction was allowed to come to rt while maintaining the Ar atmosphere. The mixture was filtered *via* cannular filtration and washed with dried dichloromethane (200 mL, 2×60, 1×80 mL). The reaction product was dried *in vacuo* for several hours and stored in an Ar filled glovebox. The collected filtrates were reduced *in vacuo* to afford 10.521 g, 73.5 mmol unreacted 4‐methyl‐5‐thiazole ethanol. Accordingly, **IM‐NHC5** was obtained as pale‐yellow beads with a pre‐catalyst loading of 3.36 mmol ⋅ g^−1^. Apart from gravimetric determination of the pre‐catalyst loading, several ICP samples were acquired and based on ICP measurements, the loading was determined to be 3.24 mmol ⋅ g^−1^ which is in good agreement with the experimentally obtained value. For all experiments carried out in this work, pre‐catalyst loading as determined by ICP was used.

### General Procedure of a Homogeneously Catalysed Experiment using Various Pre‐Catalysts

To a 10 mL thick‐walled micro‐wave tube with stir bar was added pre‐catalyst, base, acetaldehyde, ethanol as solvent (if described) and mesitylene as internal standard. The tube was sealed and the reaction was carried out in an *AntonPaar Monowave* 450 microwave reactor for a given amount of time at a given temperature. After the reaction, the tube was carefully opened and analysed by GC‐FID. Response factors of acetoin and acetaldehyde were determined by calibration using mesitylene as internal standard.

### General Procedure of Batch (Recycling) Experiments using IM‐NHC5 Pre‐Catalyst and Base

To a thick‐walled glass pressure tube (*ACE*, 20 mL) was added pre‐catalyst **IM‐NHC5** (119.8 mg, 3.24 mmol ⋅ g^−1^, 0.4 mmol, 1.0 eq, 2.0 mol%) as well as Na_2_CO_3_ (53.4 mg, 0.5 mmol, 1.3 eq, 2.6 mol%) inside an Ar‐filled glovebox. Outside of the glovebox was added either an acetaldehyde and mesitylene containing stock‐solution in ethanol or acetaldehyde, ethanol and mesitylene separately in Ar counterflow. The pressure tube was then tightly closed in Ar counterflow and heated in an oil‐bath and stirred for the indicated time. Afterwards the mixture was allowed to come to room temperature before submerging in an ice‐bath for 15 minutes. The contents of the pressure tube were subjected to GC‐FID analysis. Response factors of acetoin and acetaldehyde were determined by calibration using mesitylene as internal standard.

### General Procedures of a Continuous Flow Reaction

A stock solution for a continuous reaction was prepared by mixing acetaldehyde, ethanol and mesitylene in appropriate amounts. Inside an Ar filled glovebox, the desired amount of pre‐catalyst **IM‐NHC5** and Na_2_CO_3_ was weighed, based on *V*(CSTR)= 30.6 mL and the content of the previously prepared stock solution.

To start the reaction, the heating cone was first preheated to the desired reaction temperature of 70 °C. Meanwhile, pre‐catalyst **IM‐NHC5** and Na_2_CO_3_ were added to the dry reactor and the reactor was manually filled with stock solution (20 mL). The reactor was sealed and the pump was started to fill the reactor. The flow rate was first set to *V̇*(stock)=2.5 mL ⋅ min^−1^. Once the reactor is filled, the pump rate is set to the desired value of *V̇*(stock)=0.475 mL ⋅ min^−1^ and the reactor is placed in the preheated heating cone. The first reaction solution is taken when the first product droplet leaves the system, marking the start of the experiment. A sample is taken every 15 minutes for a period of 4 minutes.

At the end of the experiment, the reactor is removed from the heating cone and cooled in a water bath. Once the reactor has cooled to rt, the pump is switched off and the catalyst is removed from the reactor for further characterisation. The description of the continuous flow reactor can be found in the SI.

### Solid‐State NMR

The solid‐state NMR experiments were recorded using 4.0 mm double resonance and 3.2 mm triple‐resonance Bruker probes at a Bruker Avance III HD 11.7 T spectrometer. The spectra were processed with the software Topspin (version 3.6.4 & 4.1., Bruker Biospin). The experimental parameters were optimized directly on the samples where possible. Solid adamantane and ^13^C, ^15^N labelled glycineethylester have been employed for setting up experiments for samples with low signal‐to‐noise ratios in the corresponding spectra. ^13^C spectra were referenced to TMS using the methylene resonance of adamantane as an external standard (38.56 ppm). ^15^N spectra were referenced with respect to liquid NH_3_ using NH_4_Cl set to 39.3 ppm.^[38]^ The spectra have been recorded at a temperature of 280 K (Bruker BCU‐II temperature). For the experimental details see the supporting information.

### Ab Initio Calculations of ^15^N Magnetic‐Shielding Tensors

The initial geometries for 4‐methyl‐5‐thiazole ethanol and NHC5 were manually built with the software Avogadro.[^42^, ^43^] Geometry optimization of the initial structures was carried out with the program package TURBOMOLE[^44^, ^45^] (version 7.5.1) on a DFT meta‐GGA (r^2^SCAN‐3c)^[46]^ level of theory, applying the D4^[47]^ dispersion correction and a def2‐*m*TZVPP^[48]^ basis set. For all calculations the default SCF energy convergence criterion of 10^−7^ *E_h_* was used together with an integration grid of *m*4^[49]^ and the resolution of identity (RI)[^49^, ^50^] approximation.

The calculations of the ^15^N magnetic shielding tensors were based on the optimized geometries and carried out in the ORCA[^51^, ^52^] program package (version 5.0.4) with the Gauge Including Atomic Orbital^[53]^ (GIAO) method employing the PBE0^[54]^ functional and the triple‐ξ pcSseg‐2^[55]^ basis set with resolution of identity for Coulomb and exchange integrals (RIJK)^[56]^ and auxiliary basis sets automatically generated with the *AutoAux* ^[57]^ algorithm.

## Conflict of Interests

The authors declare not conflict of interest.

1

## Supporting information

As a service to our authors and readers, this journal provides supporting information supplied by the authors. Such materials are peer reviewed and may be re‐organized for online delivery, but are not copy‐edited or typeset. Technical support issues arising from supporting information (other than missing files) should be addressed to the authors.

Supporting Information

## Data Availability

The data that support the findings of this study are available in the supplementary material of this article.
