# Supplementary material for: Catalytic Upgrading of Acetaldehyde to Acetoin Using a Supported N‐Heterocyclic Carbene Catalyst
Source: ChemSusChem. 2024 Aug 8;17(22):e202400647. doi: 10.1002/cssc.202400647 (PMC11587688; doi:10.1002/cssc.202400647)
Supplement: Supplementary file 1 — Supporting Information [file CSSC-17-e202400647-s001.pdf]

ChemSusChem

Supporting Information

# Catalytic Upgrading of Acetaldehyde to Acetoin Using a Supported N-Heterocyclic Carbene Catalyst

Maurice Belleflamme, Jerome Hommes, Riza Dervisoglu, Ettore Bartalucci, Thomas Wiegand, Anna Katharina Beine, Walter Leitner, and Andreas J. Vorholt*

## Supporting Information

### Synthesis of NHC6

**NHC6** was prepared according to a procedure describe in literature. To a 10 mL Schlenk tube was added 2,4,6-trimethylbenzyl chloride (0.3408 g, 2.02 mmol, 1.0 eq) as well as 4-methyl-5-(2-hydroxyethyl)-thiazole (0.3213 g, 2.24 mmol, 1.1 eq). MeCN (2.0 mL) was added as solvent and the reaction was stirred for 24 h at 80 °C. Afterwards, the volatiles were removed *in vacuo* and the residual white fluffy solid was washed with MeCN and a recrystallisation from hot MeCN was carried out. The product was recovered as white, fluffy solid (0.3221 g, 1.033 mmol, 51.1%, $T$mp = 172.0 °C). Further analytical data can be found in the publication by *Yamazaki et al.*[1]

### Description of the continuous stirred tank reactor (CSTR) miniplant

In the continuous stirred tank reactor setup (**Figure S 1**), the stock solution is pumped from the cooled storage tank (E-1) through a 1/8" PFA capillary by *Shimadzu LC-10AD* HPLC pump (E-2), through a 1/16" stainless steel capillary to the reactor (E-3). The reactant solution is fed into to the stirred tank reactor (E-4), which consists of a stainless steel autoclave with a volume of $V$ = 30.6 mL. The autoclave is heated by a stirring-hot plate and a heating cone. The product solution is passed through a riser tube consisting of a 1/8" PFA capillary to the back-pressure regulator (V-1) (BPR). The solution is then passed through a 1/8" PFA capillary, which is cooled in an ice bath (E-5), from the backpressure valve to the collection vessel (E-6), where it is collected and samples can be withdrawn.

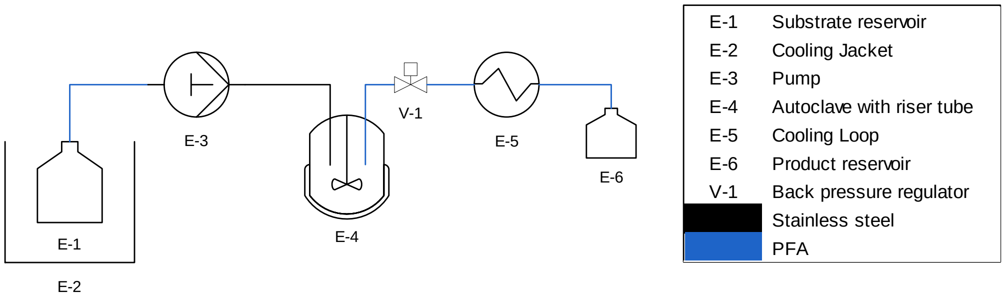


**Figure S 1:** Flow diagram of the continuous tsirred tank reactor.

### Experimental determination of residence time

The setups shown in **Figure S 2** were used to investigate the residence times. The general setup was modified so that a 6/2-way valve (V-1), from *Shimadzu* model *FCV-20AH2*, was installed between the pump (E-2) and the reactor (E-6). At the 6/2-way valve, syringe (E-3) was used to introduce the tracer into the sample loop (E-5), with a sample volume of 100 µL. Ink was used as tracer, which was first evaporated and dissolved again with MilliQ water. Excess solution was collected in the collection container (E-4). By switching the 6/2-way valve, the flow was directed through the sample loop and thus the tracer solution was fed into the stirred tank reactor (E-6) and further into the backpressure regulator (V-2). To detect the tracer, the UV-Vis detector of an HPLC (E-7) was used. The solution was then collected in the collection vessel (E-8).

All measurements were performed under reaction temperatures. For this purpose, first the heating of the reactor was set to the desired temperature and solution was pumped through the system until it reached the collection vessel. Then, the system was additionally purged for 15 min. After the time elapsed, the residence time measurement was started by switching the flow in the 6/2-way valve through the sample loop and starting the measurement in the LabSolutions program.

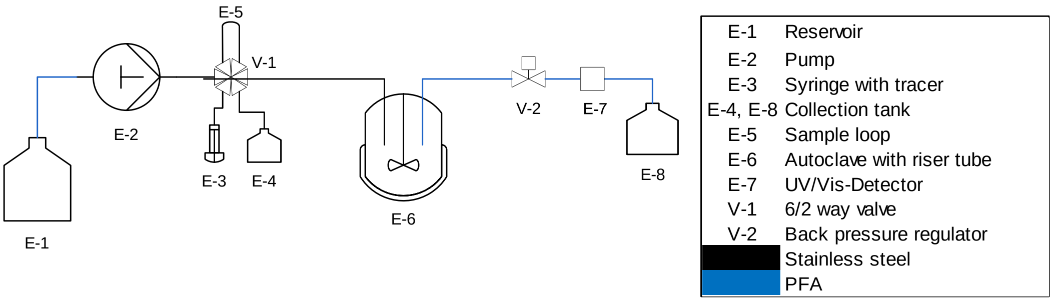


**Figure S 2:** Flow diagram of the continuous stirred tank reactor set-up for residence time measurements.

## Calculation of residence time

Residence times were measured at flows ranging from 0.05 to 1.00 mL·min$^{-1}$ and at reaction temperatures to characterize the system. A shock marker with ink was used to determine the residence time. For this purpose, a 6/2-way valve and a UV-Vis detector were installed in the setup. The flow diagrams for the determination of the residence time is shown in the supporting information. To determine the average residence time for the CSTR, the exponential decay of the UV-Vis signal was investigated and an exponential fit equation **(1)** was used to fit the signal.[2]

$$f(x) = y_0 + A \cdot e^{-\frac{x}{\tau}} \quad \textbf{(1)}$$

To determine the required flow for an arbitrary residence time based on the measured data, the formula shown in equation **(2)** was obtained by a power regression.

$$\dot{V} = 26.348 \cdot \tau^{-0.839} \quad \textbf{(2)}$$

## Results of various catalyst screening experiments

**Table S 1:** Catalytic results of the NHC and base catalysed acetaldehyde addition using an NHC catalyst in polar protic solvents.[a]

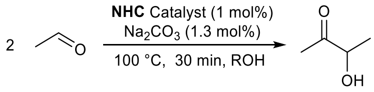


| Entry | NHC catalyst | solvent | *Y*(acetoin)[b] / % | ∑*Y*(side products)[b,c] / % |
|---|---|---|---|---|
| 1 | **NHC1** | EtOH | 98 | - |
| 2 | **NHC7** | EtOH | 15 | 7 |
| 3 | **NHC1** | MeOH | 38 | 8 |
| 4[d] | **NHC7** | MeOH | 9 | 3 |

[a] reaction conditions: *V*(acetaldehyde) = 1.0 mL, *n*(acetaldehyde) = 18 mmol, *V*(solvent) = 5.0 mL, *c*(NHC) = 1.0 mol%, *c*(Na2CO3) = 1.0 mol%, *T* = 100 °C, *t* = 30 min, 800 rpm. [b] determined by GC-FID using mesitylene as an internal standard. [c] total yield aldol products. [d] *V*(MeOH) = 2.0 mL.

**Table S 2:** Catalytic results of the **NHC6** and $Na_2CO_3$ catalysed acetaldehyde addition in cyclohexane as solvent.[a]

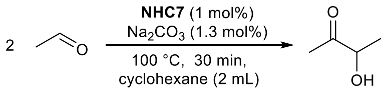


| Entry | Phase | *Y*(acetoin)[b] / % |
|---|---|---|
| 1 | organic | 1 |
| 2 | product | 73 |
| 3[c] | - | 73 |

[a] reaction conditions: *V*(acetaldehyde) = 2.0 mL, *n*(acetaldehyde) = 36 mmol, *c*(NHC) = 1.0 mol%, *c*($Na_2CO_3$) = 1.3 mol%, *V*(cyclohexane) = 2.0 mL, *T* = 100 °C, *t* = 30 min, 600 rpm. [b] determined by GC-FID using mesitylene as an internal standard. [c] neat reaction conditions without additional solvent.

**Table S 3:** Catalytic results of the **NHC5**/$Na_2CO_3$ catalysed acetaldehyde addition at different temperatures and reaction times.[a]

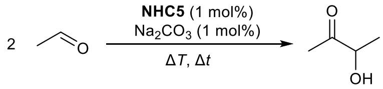


| Entry | *T* / °C | *t* / h | *Y*(acetoin)[b] / % | *TON* (acetoin) |
|---|---|---|---|---|
| 1 | 50 | 1 | 15 | 7 |
| 2 | 50 | 3 | 72 | 35 |
| 3 | 60 | 1 | 66 | 32 |
| 4 | 60 | 3 | 89 | 43 |
| 5 | 70 | 1 | 87 | 42 |
| 6 | 70 | 3 | 88 | 42 |
| 7[c] | 70 | 1 | 95 | 46 |
| 8[c,d,e] | 70 | 1 | 84 | 40 |
| 9[c,d] | 70 | 1 | 95 | 46 |

[a] reaction conditions: *V*(acetaldehyde) = 2.0 mL, *n*(acetaldehyde) = 36 mmol, *c*(**NHC1**) = 1.0 mol%, *c*($Na_2CO_3$) = 1.0 mol%, not additional solvent. [b] determined by GC-FID using mesitylene as an internal standard. [c] *c*($Na_2CO_3$) = 1.3 mol%. [d] experiment carried out in an autoclave. [e] Mesitylene added after completion of the reaction.

**Table S 4:** Results of the **IM-NHC5**/$Na_2CO_3$ catalysed acetaldehyde self-addition in EtOH as solvent.[a]

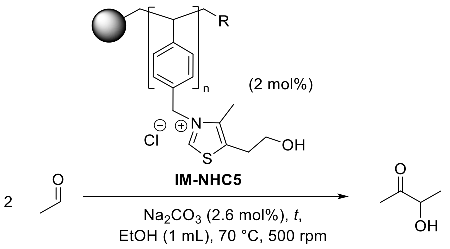


| Entry | *t* / h | *Y*(acetoin)[b,c] / % | *TON* (acetoin) |
|---|---|---|---|
| 1 | 16 | 93 | 50 |
| 2 | 8 | 95 | 48 |
| 3 | 2 | 86 | 46 |

[a] reaction conditions: *V*(acetaldehyde) = 1.0 mL, *n*(acetaldehyde) = 18 mmol, *V*(EtOH) = 1.0 mL, *c*(**IM-NHC5**) = 2 mol%, *c*($Na_2CO_3$) = 2.6 mol%, *T* = 70 °C, 500 rpm. [b] As determined by GC-FID using mesitylene as internal standard [c] acetoin yields including the yield of dimeric acetoin.

**Table S 5:** Results of the **IM-NHC5**/base catalysed acetaldehyde self-addition in EtOH as solvent using different bases as well as different activation strategies.[a]

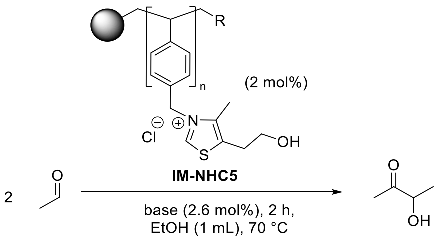


| Entry | Base | *Y*(acetoin)[b,c] / % | ∑*Y*(side-products)[b,d] / % | *TON* (acetoin) |
|---|---|---|---|---|
| 1 | $Na_2CO_3$ | 75 | 3.5 | 38 |
| 2 | $Na_2CO_3$[e] | <0.1 | 34 | - |
| 3 | DEA | 7 | 53 | 4 |
| 4 | NaO*t*Bu | 42 | < 0.1 | 23 |
| 5 | KO*t*Bu | 55 | < 0.1 | 30 |
| 6 | NaOEt | 49 | < 0.1 | 28 |

[a] reaction conditions: *V*(acetaldehyde) = 1.0 mL, *n*(acetaldehyde) = 18 mmol, *V*(EtOH) = 1.0 mL, c(**IM-NHC5**) = 2 mol%, c($Na_2CO_3$) = 2.6 mol%, *T* = 70 °C, *t* = 2 h [b] As determined by GC-FID using mesitylene as internal standard [c] acetoin yields including the yield of dimeric acetoin [d] side products include 1,1-diethoxyethane, paraldehyde and others. [e] a saturated solution of $Na_2CO_3$ in EtOH (dry, absolute) was prepared prior to the reaction and used as solvent *V*($Na_2CO_3$ saturated EtOH) = 1.0 mL.

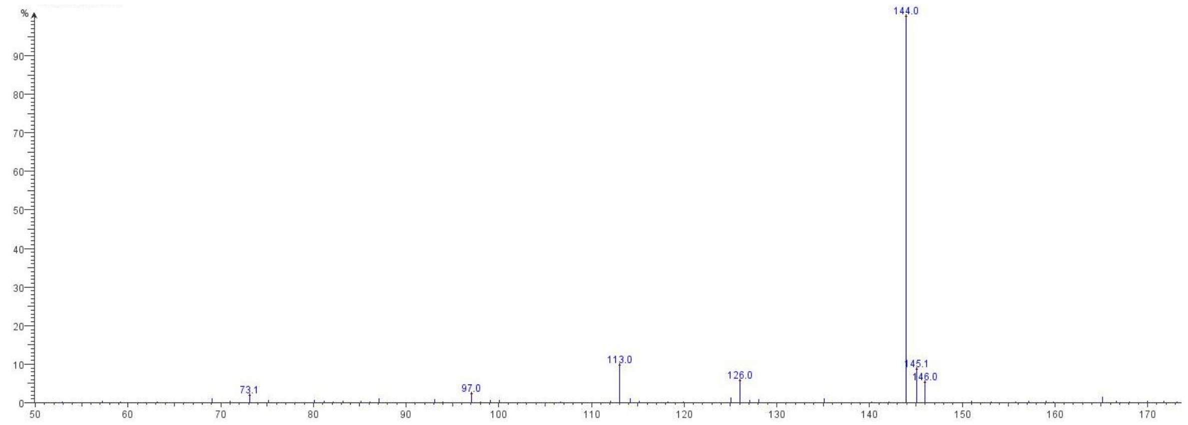


**Figure S 3:** ESI-MS spectrum in the 50 to 175 *m/z* region of an experiment where **IM-NHC5**/$Na_2CO_3$ was heated in EtOH (*V* = 5.0 mL) for a duration of 2 h at 70 °C.

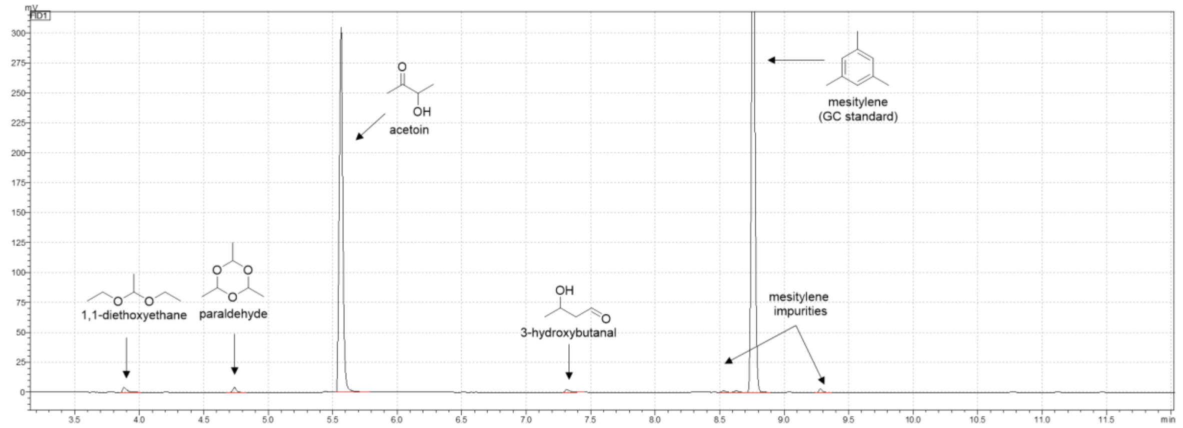


**Figure S 4:** Close-up of a GC chromatogram of a typical continuous flow reaction, using the **IM-NHC5**/$Na_2CO_3$ reaction system in the acetaldehyde self-addition shown in **Figure 3**. Compounds were assigned to peaks using GC-MS measurements with the same Rtx1701 column.

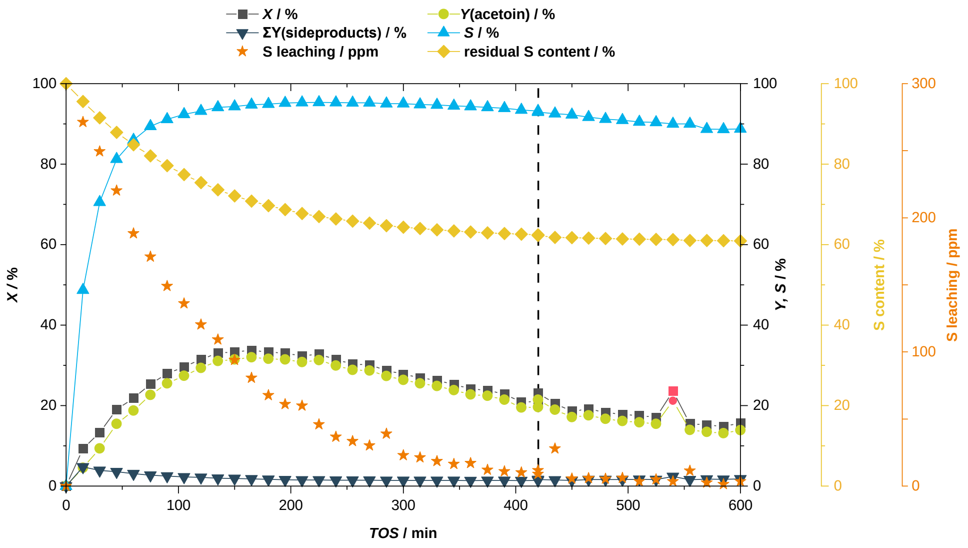


**Figure S 5:** Results of the continuous flow acetaldehyde self-addition in a CSTR setup over a total of 10 h TOS. Graph shows the conversion *X*, yield *Y* and selectivity *S* towards acetoin over TOS as well as the calculated residual Sulphur content (%) of the catalyst and the measured leaching of S (ppm) for samples taken every 15 minutes for a duration of 4 minutes. Reaction conditions: stock-solution containing acetaldehyde (150 mL, 118.6 g, 2.7 mol), EtOH (350 mL, 274.0 g, 5.9 mol) and mesitylene (29.0 mL, 24.93 g), $\dot{V}$ (stock) = 0.475 mL·min$^{-1}$, *m*(**IM-NHC5**) = 1.0445 g (3.24 mmol·g$^{-1}$, 3.38 mmol NHC, 2 mol%) *m*($Na_2CO_3$) = 467.3 mg (4.49 mmol, 2.6 mol%), *T* = 70 °C, 1000 rpm. Mass-balances Σ*Y* > 89% were obtained. Dotted vertical line at 420 min TOS, indicates that the reaction was terminated over-night and re-started the next day. Red dot/square indicate outlier values in the determination of *X* and *Y*.

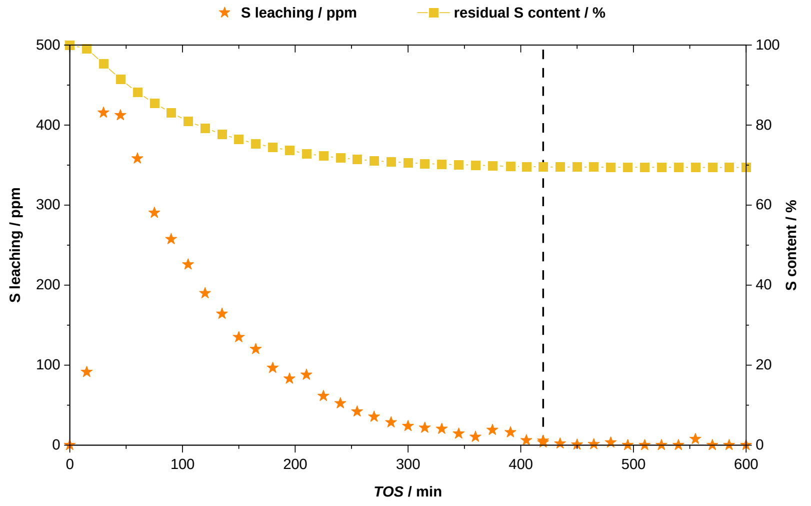


**Figure S 6:** Results of the continuous flow blind experiment in absence of acetaldehyde. The measured (XRF) leaching of S was used to construct the plot of residual S content over TOS and serves as guide to the eye. Reaction conditions: stock-solution containing etanol and mesitylene, $\dot{V}$(stock) = 0.475 mL·min$^{-1}$, *m*(**IM-NHC5**) = 1.7305 g (3.24 mmol·g$^{-1}$, 5.6 mmol NHC, 1.0 eq) m($Na_2CO_3$) = 776.6 mg (7.33 mmol, 1.3 eq), *T* = 70 °C, 1000 rpm. Dotted vertical line at 420 min TOS, indicates that the reaction was terminated over-night and re-started the next day.

## Solid-state NMR Spectroscopy and DFT calculations

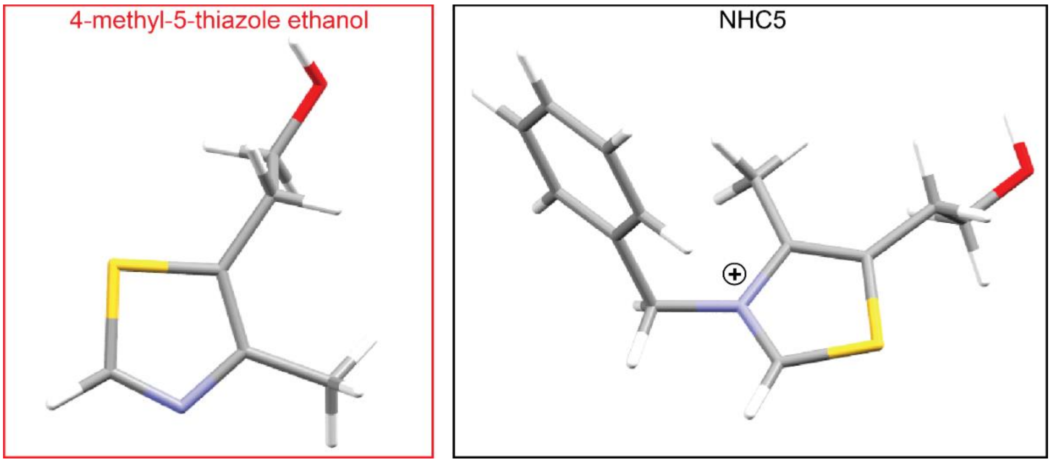


**Figure S 7**: DFT meta-GGA (r2SCAN-3c)[3] level of theory, applying the D4[4] dispersion correction and a def2-mTZVPP[5] basis set ooptimized structures used for the calculations of GIAO[6] $^{15}$N magnetic-shielding tensors for 4-methyl-5-thiazole ethanol and **NHC5**.

**Table S 6:** Comparison of calculated and experimental absolute $^{15}$N isotropic chemical-shift difference (|Δδ|) between 4-methyl-5-thiazole ethanol and **NHC5**.

| Molecule | Experimental \|Δδ($^{15}$N)/ ppm | Calculated \|Δδ($^{15}$N)\|/ ppm |
|---|---|---|
| 4-methyl-5-thiazole ethanol<br>**NHC5** | 108.2 | 122.9 |

**Table S 7:** Overview of 13C and 15N MAS NMR experimental parameters performed with in a Bruker 4 mm double-resonance HX probe.

| **Sample** | **4-methyl-5-thiazole ethanol** | **4-methyl-5-thiazole ethanol** | **NHC5** | **NHC5** | **IM-NHC5** | **IM-NHC5** | ***Merrifield* polymer resin** |
|---|---|---|---|---|---|---|---|
| **Experiment** | $^{13}$C MAS | $^{15}$N INEPT MAS | $^{1}$H-$^{13}$C CP MAS | $^{1}$H-$^{15}$N CP MAS | $^{1}$H-$^{13}$C CP MAS | $^{1}$H-$^{15}$N CP MAS | $^{1}$H-$^{13}$C CP MAS |
| $\nu_r$ / kHz | 14.0 | 8.0 | 14.0 | 14.0 | 14.0 | 14.0 | 14.0 |
| $B_0$ / T | 11.7 | 11.7 | 11.7 | 11.7 | 11.7 | 11.7 | 11.7 |
| Transfer I | Direct polarization | H-N INEPT | H-C CP | H-N CP | H-C CP | H-N CP | H-C CP |
| $\nu_1(^1H)$ / kHz | - | - | 60 | 60 | 60 | 60 | 60 |
| $\nu_1(X)$ / kHz | - | - | 51.5 | 48 | 51.5 | 48 | 51.5 |
| Shape | - | - | | | Tangent shape[7] | | |
| $^{13}$C/$^{15}$N carrier / ppm | 97.8 | 200 | 100 | 200 | 100 | 200 | 100 |
| CP contact time / ms | - | - | 2.0 | 5.0 | 2.0 | 10.0 | 2.0 |
| $t_2$ increments | 59990 | 16384 | 2048 | 2048 | 2048 | 2048 | 2048 |
| Sweep width ($t_2$) / ppm | 795 | 1006 | 397 | 1006 | 397 | 986 | 397 |
| Acquisition time ($t_2$) / ms | 299.9 | 160.5 | 20.5 | 20.0 | 20.5 | 20.5 | 20.5 |
| $^{1}$H Spinal64/Waltz64 decoupling / kHz | 62.5 | 5.0 | 90.0 | 90.0 | 83.0 | 83.0 | 83.0 |
| Recycle delay / s | 10.0 | 5.0 | 3.0 | 3.0 | 5.0 | 5.0 | 5.0 |
| Number of scans | 16 | 1024 | 512 | 42000 | 512 | 56160 | 512 |
| Measurement time / h | 0.04 | 1.42 | 0.43 | 25 | 0.71 | 78 | 0.71 |

**Table S 8:** Overview of $^{13}C$ and $^{15}N$ MAS NMR experimental parameters performed with 3.2mm HXY probe.

| Sample | AVG-MB-455-42 Figure 5 | AVG-MB-455-43 Ground Figure 5 | AVG-MB-472-64 Figure 8 | AVG-MB-474-42 Figure S 6 | NHC5 Figure 1 | NHC5 Figure 1 |
|---|---|---|---|---|---|---|
| **Experiment** | $^{1}H$-$^{13}C$ CP MAS | $^{1}H$-$^{13}C$ CP MAS | $^{1}H$-$^{13}C$ CP MAS | $^{1}H$-$^{13}C$ CP MAS | $^{1}H$-$^{15}N$ CP MAS | $^{1}H$-$^{13}C$ CP MAS |
| $\nu_r$ / kHz | 14.0 | 14.0 | 14.0 | 14.0 | 14.0 | 14.0 |
| $B_0$ / T | 11.7 | 11.7 | 11.7 | 11.7 | 11.7 | 11.7 |
| **Transfer I** | **H-C CP** | **H-C CP** | **H-C CP** | **H-C CP** | **H-N CP** | **H-C CP** |
| $\nu_1(^{1}H)$ / kHz | 60 | 60 | 60 | 60 | 60 | 60 |
| $\nu_1$(X) / kHz | 39 | 39 | 39 | 39 | 36 | 39 |
| Shape | | | | Tangent shape[7] | | |
| $^{13}C$/$^{15}N$ carrier / ppm | 100 | 100 | 100 | 100 | 200 | 100 |
| CP contact time / ms | 2 | 2 | 2 | 2 | 5 | 2 |
| $t_2$ increments | 2048 | 2048 | 2048 | 2048 | 2048 | 2048 |
| Sweep width ($t_2$) / ppm | 397 | 397 | 397 | 397 | 1007 | 397 |
| Acquisition time ($t_2$) / ms | 20.5 | 20.0 | 20.5 | 20.5 | 20.5 | 20.5 |
| $^{1}H$ Spinal64/Waltz64 decoupling / kHz | 90 | 90 | 90 | 90 | 90 | 90 |
| Recycle delay / s | 3.0 | 3.0 | 3.0 | 3.0 | 3.0 | 3.0 |
| Number of scans | 12288 | 42000 | 2048 | 56160 | 10240 | 2048 |
| Measurement time / h | 10 | 25 | 2 | 8 | 8 | 2 |

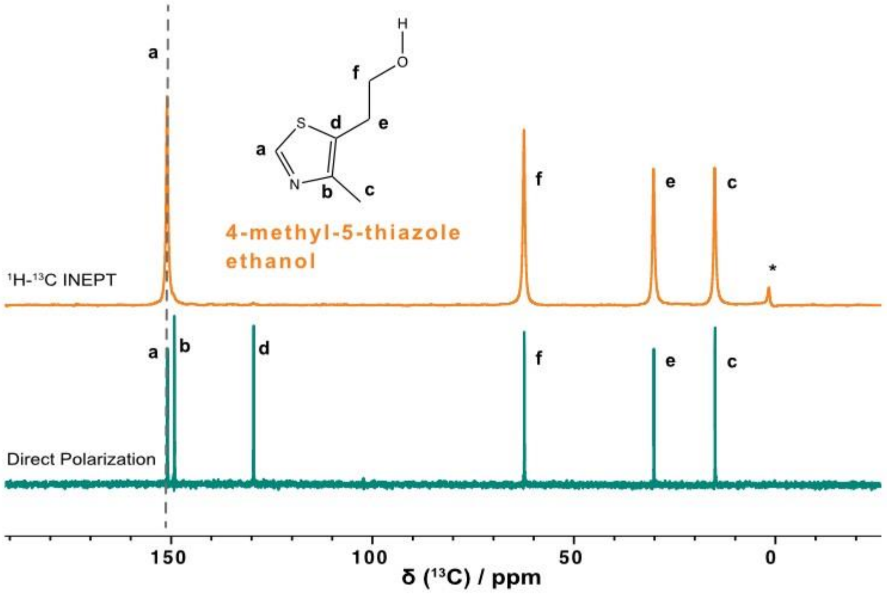


**Figure S 8:** $^{13}C$ direct polarization (green) and $^{1}H$-$^{13}C$ INEPT (orange) spectra of 4-methyl-5-thiazole ethanol. Methine, methylene and methyl carbon atoms (a, c, e, f) can be distinguished from quaternary carbon atoms (b, d). * denotes a peak of the silicone plug used for sealing the liquid sample inside the MAS rotor.

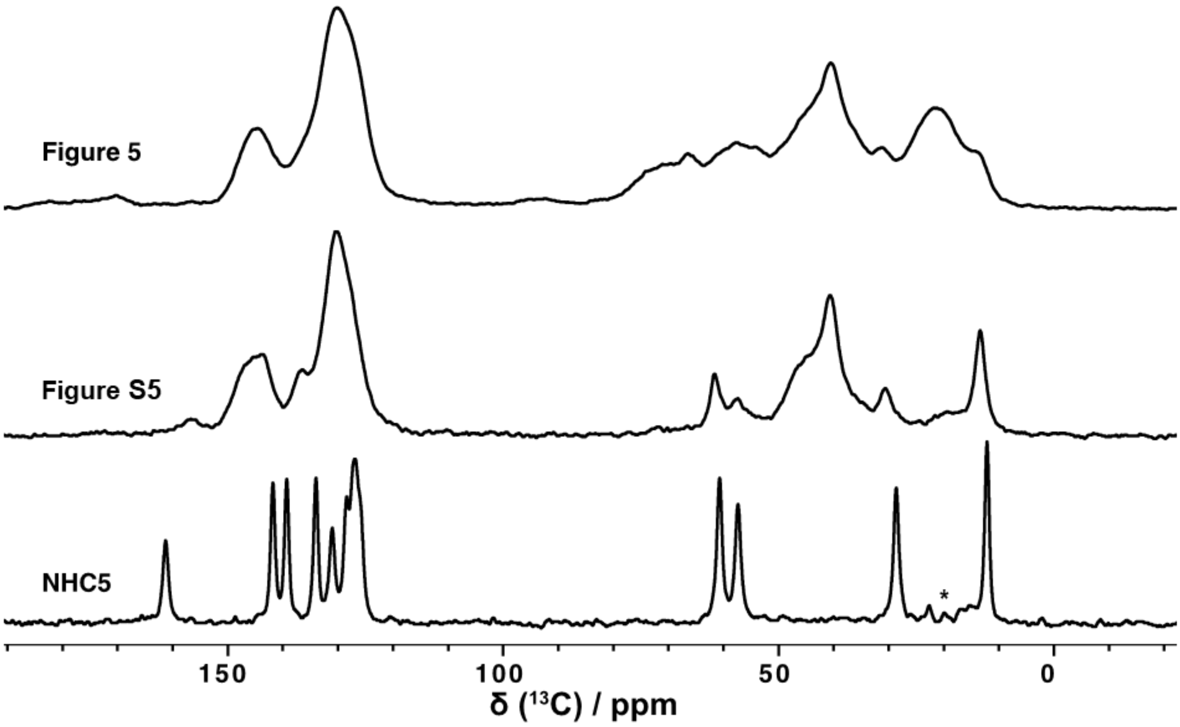


**Figure S 9:** $^{1}H$-$^{13}C$ CP NMR spectra of various spent **IM-NHC5** catalysts from **Figures 5**, **S 5** as well as the **NHC5** from **Figure 1**. 160 ppm peak is observed for the leaching of the 4-methyl-5-thiazole ethanol from the polymer. All spectra have been rescorded at 11.7 T and 14.0 kHz MAS frequency. * denotes MAS side band.

## References


[1] K. Terashima, T. Kawasaki-Takasuka, T. Agou, T. Kubota, T. Yamazaki, *Organic & Biomolecular Chemistry* **2020**, *18*, 4638.

[2] M. Baerns, A. Behr, H. Hofmann, J. Gmehling, U. Onken, A. Renken, K.-O. Hinrichsen, R. Palkovits, *Technische chemie*, Wiley-VCH Verlag GmbH & Co. KGaA, **2013**.

[3] S. Grimme, A. Hansen, S. Ehlert, J. M. Mewes, *J. Chem. Phys.* **2021**, *154*, 064103.

[4] E. Caldeweyher, S. Ehlert, A. Hansen, H. Neugebauer, S. Spicher, C. Bannwarth, S. Grimme, *J. Chem. Phys.* **2019**, *150*, 154122.

[5] F. Weigend, R. Ahlrichs, *Phys. Chem. Chem. Phys.* **2005**, *7*, 3297.

[6] J. R. Cheeseman, G. W. Trucks, T. A. Keith, M. J. Frisch, *J. Chem. Phys.* **1996**, *104*, 5497.

[7] S. Hediger, B. H. Meier, R. R. Ernst, *Chem. Phys. Lett.* **1995**, *240*, 449.
